# Supplementary material for: Determinants of implementing deprescribing for older adults in English care homes: a qualitative interview study
Source: BMJ Open. 2023 Nov 23;13(11):e081305. doi: 10.1136/bmjopen-2023-081305 (PMC10668129; doi:10.1136/bmjopen-2023-081305)
Supplement: Supplementary data [file bmjopen-2023-081305supp001.pdf]

STOPPING

Understanding stakeholders' perspectives on implementing deprescribing in care homes (STOPPING)

TOPIC GUIDE WITH CARE HOME STAFF AND HEALTHCARE PROFESSONALS

Introduction

We are here today to talk about your experiences with and views of deprescribing (stopping a medication or reducing its dosage) in care homes. The purpose is to get your perceptions of how reducing or stopping some medicines would work in a care home setting so we can identify the key barriers, facilitators and contextual factors influencing it. I am not here to share information, or to give you my opinions. Your perceptions are what matter. There are no right or wrong or desirable or undesirable answers. You can disagree with each other, and you can change your mind. I would like you to feel comfortable saying what you really think and how you really feel.

As you know everything is confidential.

Topics

Knowledge & beliefs about the intervention

What do you think about reducing or stopping some medicines for people living in care homes?

Probes. Why do you think that? Do you have any concerns? Are there any benefits?

Do you think reducing or stopping some medicines will be beneficial in your care home?

Probes. Why or Why not? Do you have any concerns?

Relative advantage

Do you think are the advantages of reducing or stopping some medicines for people living in care homes compared to continuing their medicines?

Probes. Why? Is that your experience? Anything else?

Do you think are the disadvantages of reducing or stopping some medicines for people living in care homes compared to continuing their medicines?

Probes. Why? Is that your experience? Anything else?

Tension for change

Is there a strong need for reducing or stopping some medicines?

Probes. Why or why not? Do others see a need for it?

How essential is this reducing or stopping some medicines to meet the needs of the individuals served by your care home?

How do you feel about current programs/practices/process that are available?

Probes. What do they do well? To what extent do current programs fail to meet existing needs?

Complexity

How complicated is it to reduce or stop some medicines for care home residents?

Probes. Please consider the following aspects: duration, scope, intricacy and number of steps involved

STOPPING

Trialability

What do you think about trying to stop or reduce a medicine (or medicines) for a short period?

Probes. What would that be like? Would it be helpful? Harmful? Why or why not?

Evidence strength & quality

What kind of information or evidence are you aware of that shows whether or not the reducing or stopping some medicines will work in your care home?

Probes. What evidence have you heard about? Practice guidelines? Published literature? Co-workers? Other care homes? How does this knowledge affect your view?

What kind of supporting evidence or proof is needed about the effectiveness of reducing or stopping some medicines to get staff on board?

Probes. Co-workers? Administrative leaders?

Planning

What needs to be done beforehand so that reducing or stopping medicines can happen in care homes?

Probes. Why? Is that your experience? What steps should be included? Anything else? What is your role in the planning?

Engaging

What do you think the role of the care home and its staff should be in reducing and stopping some medicines?

Probes. How would that like look? To what extent?

What do you think the role of the GPs should be?

Probes. How would that like look? To what extent?

What do you think the role of the pharmacist should be?

Probes. How would that like look? To what extent?

Is there anyone else who should be involved?

Probes. How would that like look? To what extent?

Reflecting & Evaluating

What do you think should be the outcome of reducing or stopping some medicines for people living in care homes?

Probes. Why? Is that your experience? Anything else?

What should happen after reducing or stopping some medicines?

Probes. What information should be collected? How often? Feedback given?

Communication & Network

How do you know when there have been changes to the resident’s medicines?

Probes. What gets in the way of knowing? Can this be improved? If so how?

STOPPING

Can you describe your working relationships with your colleagues?

Probes. With colleagues in your care home? With colleagues in other care homes? Can you tell me a story about a time you needed to work with others to solve a problem? Or to implement reducing to stopping a resident's medicines?

Do you understand why there have been changes to a resident's medicines?

Probes. Why? What would aid your understanding? What stops you from understanding? How could this be communicated better?

Cosmopolitanism

What kind of information sharing do you have with other professionals and organisations outside your care home, either related to the medicines, or more generally?

Probes. What professional or care home networking do you engage in? Listservs? Local or national conferences? Trainings?

Executing & Personal Attributes

Please tell me about any experiences that you may have with stopping or reducing some of the residents' medicines.

Probes. How did it go? What happened? When been done well? When has it not gone so well? What would help work better in the future? Why was that the case? Was anything done by the care home staff that helped this experience? Or impeded it? What problems or issues did you experience, if any?

Available resources

Do you expect to have sufficient resources to implement and administer reducing or stopping some medicines?

Probes. [If Yes] What resources are you counting on? Are there any other resources that you received, or would have liked to receive? What resources will be easy to procure?
[If no] What resources will not be available?

Cost

What are the costs associated with reducing or stopping a resident's medicines?

Probes. What costs will be incurred? What cost were considered when deciding to implement?

Patient needs & resources

How do the residents' needs and preferences get included in decisions to change their medicines?

Probes. Why were they included or not included? How 'in touch' were the people involved? Were they prioritised over other things? Has anything been altered or changed to meet the resident's needs? What would you like your input to be?

How would you feel about stopping or reducing some of the resident's medicines?

Probes. Is there anything that would prevent you? Why?

Self-efficacy

Do you think you can question the amount of medicines that residents are taking?

STOPPING

Probes. How confident are you to do this? Would you do it? Have you? Why or Why not?

Closure

Is there any other information regarding your experiences or opinions that you think would be useful for me to know?

Thank you very much for meeting with me. Your time is very much appreciated and your comments have been very helpful.

STOPPING

Understanding stakeholders' perspectives on implementing deprescribing in care homes (STOPPING)

INTERVIEW TOPIC GUIDE WITH CARE HOME RESIDENT

Introduction

Good morning/afternoon. My name is Krystal Warmoth. I am going to ask you some questions about reducing or stopping some prescription medicines. For example, someone may need to stop taking a medicine that may no longer be providing benefit or has side effects (like falling) as they get older, or reducing the dose of a medication so that the person doesn't have to take as many tablets each day. We still don't really know how to make it work in real-life settings, like care homes. In this project, we aim to study how reducing or stopping some medicines can be best done in care homes, considering different views and settings.

We are not wanting to change your medicines, we are just wanting to chat with you to understand what happens at the moment, in terms of how your medicines may or may not be stopped or reduced. And discuss your thoughts on this happening, and how we could one day, make sure it's done well.

Again, I want to remind you that your responses will be kept confidential and you do not have to answer any question if you do not wish to. You can also stop the interview at any time.

Before we start, I want to check that you are happy to be interviewed and for the interview to be recorded.

Interview

1. *To start, I want to know what your opinions are about reducing or stopping some medicines. What do you think about the idea of reducing or stopping some medicines?* ***Knowledge & beliefs***

   Probes. Why do you think that? Do you have any concerns? Are there any benefits?

2. *Do you think there are the* advantages of reducing or stopping some medicines*?* ***Knowledge & beliefs & Relative advantage***

   Probe. Why or Why not? Do you think are the disadvantages? Why or Why not?

3. *What do you think about trying to stop or reduce a medicine (or medicines) for a short period of time?* ***Trialability***

   Probes. What would that be like? Who would lead it? Would it be helpful? Harmful? Why or why not?

4. *What things will need to happen when planning to reduce or stop some medicines?* ***Planning***

   Probes. Why? Is that your experience? What steps should be included? Anything else? What is your role in the planning?

[CHECK IF THEY ARE HAPPY TO CONTINUE OR WOULD LIKE TO TAKE A BREAK]

5. *Do you think different approaches to reducing or stopping some medicines would be needed for different people?* ***Adaptability***

   Probes. Why or why not? What kinds of changes do you think would need to be made? Do you think these changes are possible? Why or Why not?

STOPPING

6. *What do you think the role of the care home and its staff should be in reducing and stopping some medicines?* ***Engaging***

   Probes. How would that like look? To what extent?

7. *What do you think the role of the GPs should be in reducing and stopping some medicines?* ***Engaging***

   Probes. How would that like look? To what extent?

8. *What do you think the role of the pharmacist should be in reducing and stopping some medicines?* ***Engaging***

   Probes. How would that like look? To what extent?

9. *Is there anyone else that needs to be involved in how to reduce or stop some medicines?* ***Engaging***

   Probes. Why? How would that like look? To what extent? What about family and friends?

[CHECK IF THEY ARE HAPPY TO CONTINUE OR WOULD LIKE TO TAKE A BREAK]

10. *What do you think should be the impact or end result of reducing or stopping some medicines for people living in care homes?* ***Reflecting & Evaluating***

    Probes. Why? Is that your experience? Anything else? What about quality of life? Side effects?

11. *Should anything else happen after someone's medicines have been reduced or stopped?* ***Reflecting & Evaluating***

    Probes. What information should be collected? How often? Feedback given?

12. *Did you know when there have been changes to your medicines?* ***Communication & Network***

    Probes. How did you know? What gets in the way of knowing?

13. *Did you understand why there were changes to your medicines?* ***Knowledge & beliefs***

    Probes. Why? What would aid your understanding? What stops you from understanding? How could this be communicated better?

[CHECK IF THEY ARE HAPPY TO CONTINUE OR WOULD LIKE TO TAKE A BREAK]

14. *Now, I would like to know about your own experiences with reducing or stopping some medicines. Please tell me about any experiences that you may have with stopping or reducing some of your medicines while you [they] have been living in the care home.* ***Executing & Personal Attributes***

    Probes. How did it go? What happened? When has it been done well? When has it not gone so well? What would help it work better in the future? Why was that the case? Was anything done by the care home staff that helped this experience? Or impeded it? What problems or issues did you experience, if any?

[IF CANNOT REMEMBER AN EXPERIENCE -> SKIP TO QUESTION 16]

[CHECK IF THEY ARE HAPPY TO CONTINUE OR WOULD LIKE TO TAKE A BREAK]

15. *Thinking back to your experience of medicines being stopped or reduced. How did your needs and preferences get included in decisions to change your medicines?* ***Patient needs & resources***

STOPPING

Probes. Why were they included or not included? How ‘in touch’ were the people involved? Were they prioritised over other things? Has anything been altered or changed to meet your needs? What would you like your input to be?

16. *Do you think you can question the amount of medicines that you are taking?* ***Self-efficacy***

Probes. How confident are you to do this? Would you do it? How would you talk to about this? Have you? Why or Why not?

17. How would you feel about stopping or reducing some of your medicines? ***Patient needs & resources***

Probes. Would you do it? Is there anything that would prevent you doing it? Why?

Closure

Is there any other information regarding your opinions or experiences that you think would be useful for me to know?

Again, I want to let you know that your medicines are not being changed based on what we have talked about. If you wish to change your medications as a result of this discussion, we will let the care home staff know so you can discuss this with your general practitioner and any other relevant people. Please do not change your medicines until you have talked with the appropriate healthcare professional.

Thank you very much for meeting with me. Your time is very much appreciated and your comments have been very helpful.

To finish, I am going to ask a few questions to get some basic information about you.

STOPPING

Understanding stakeholders' perspectives on implementing deprescribing in care homes (STOPPING)

INTERVIEW TOPIC GUIDE WITH FAMILY OR FRIEND

Introduction

Good morning/afternoon. My name is Krystal Warmoth. I am going to ask you some questions about reducing or stopping some prescription medicines. For example, someone may need to stop taking a medicine that may no longer be providing benefit or has side effects (like falling) as they get older, or reducing the dose of a medication so that the person doesn't have to take as many tablets each day. We still don't really know how to make it work in real-life settings, like care homes. In this project, we aim to study how reducing or stopping some medicines can be best done in care homes, considering different views and settings.

We are not wanting to change [INSERT RESIDENT'S NAME]'s medicines, we are just wanting to chat with you to understand what happens at the moment, in terms of how the [INSERT RESIDENT'S NAME]'s medicines may or may not be stopped or reduced. And discuss your thoughts on this happening, and how we could one day, make sure it's done well.

Again, I want to remind you that your responses will be kept confidential and you do not have to answer any question if you do not wish to. You can also stop the interview at any time.

Before we start, I want to check that you are happy to be interviewed and for the interview to be recorded.

Interview

18. *To start, I want to know what your opinions are about reducing or stopping some medicines. What do you think about the idea of reducing or stopping some medicines?* ***Knowledge & beliefs***

    Probes. Why do you think that? Do you have any concerns? Are there any benefits?

19. *Do you think there are the* advantages of reducing or stopping some medicines*?* ***Knowledge & beliefs & Relative advantage***

    Probe. Why or Why not? Do you think are the disadvantages? Why or Why not?

20. *What do you think about trying to stop or reduce a medicine (or medicines) for a short period of time?* ***Trialability***

    Probes. What would that be like? Who would lead it? Would it be helpful? Harmful? Why or why not?

21. *What things will need to happen when planning to reduce or stop some medicines?* ***Planning***

    Probes. Why? Is that your experience? What steps should be included? Anything else? What is your role in the planning?

[CHECK IF THEY ARE HAPPY TO CONTINUE OR WOULD LIKE TO TAKE A BREAK]

22. *Do you think different approaches to reducing or stopping some medicines would be needed for different people living in a care home?* ***Adaptability***

    Probes. Why or why not? What kinds of changes do you think would need to be made? Do you think these changes are possible? Why or Why not?

STOPPING

23. *What do you think the role of the care home and its staff should be in reducing and stopping some medicines?* ***Engaging***

    Probes. How would that like look? To what extent?

24. *What do you think the role of the GPs should be in reducing and stopping some medicines?* ***Engaging***

    Probes. How would that like look? To what extent?

25. *What do you think the role of the pharmacist should be in reducing and stopping some medicines?* ***Engaging***

    Probes. How would that like look? To what extent?

26. *Is there anyone else that needs to be involved in how to reduce or stop some medicines?* ***Engaging***

    Probes. Why? How would that like look? To what extent? What about family and friends?

[CHECK IF THEY ARE HAPPY TO CONTINUE OR WOULD LIKE TO TAKE A BREAK]

27. *What do you think should be the impact or end result of reducing or stopping some medicines for people living in care homes?* ***Reflecting & Evaluating***

    Probes. Why? Is that your experience? Anything else? What about quality of life? Side effects?

28. *Should anything else happen after someone's medicines have been reduced or stopped?* ***Reflecting & Evaluating***

    Probes. What information should be collected? How often? Feedback given?

29. *Did you know when there have been changes to [INSERT RESIDENT'S NAME]'s medicines?* ***Communication & Network***

    Probes. How did you know? What gets in the way of knowing?

30. *Did you understand why there were changes to [INSERT RESIDENT'S NAME]'s medicines?* ***Knowledge & beliefs***

    Probes. Why? What would aid your understanding? What stops you from understanding? How could this be communicated better?

[CHECK IF THEY ARE HAPPY TO CONTINUE OR WOULD LIKE TO TAKE A BREAK]

31. *Now, I would like to know about your own experiences with reducing or stopping some medicines. Please tell me about any experiences that you may have with stopping or reducing some of [INSERT RESIDENT'S NAME]'s medicines while they have been living in the care home.* ***Executing & Personal Attributes***

    Probes. How did it go? What happened? When has it been done well? When has it not gone so well? What would help it work better in the future? Why was that the case? Was anything done by the care home staff that helped this experience? Or impeded it? What problems or issues did you experience, if any?

[IF CANNOT REMEMBER AN EXPERIENCE -> SKIP TO QUESTION 16]

[CHECK IF THEY ARE HAPPY TO CONTINUE OR WOULD LIKE TO TAKE A BREAK]

STOPPING

32. *Thinking back to your experience of medicines being stopped or reduced. How did your needs and preferences get included in decisions to change [INSERT RESIDENT'S NAME]'s medicines?* ***Patient needs & resources***

    Probes. Why were they included or not included? How 'in touch' were the people involved? Were they prioritised over other things? Has anything been altered or changed to meet your [resident's] needs? What would you like your input to be?

33. *Do you think you can question the amount of medicines that [INSERT RESIDENT'S NAME] are taking?* ***Self-efficacy***

    Probes. How confident are you to do this? Would you do it? How would you talk to about this? Have you? Why or Why not?

34. How would you feel about stopping or reducing some of *[INSERT RESIDENT'S NAME]'s* medicines? ***Patient needs & resources***

    Probes. Would you do it? Is there anything that would prevent you doing it? Why?

Closure

Is there any other information regarding your opinions or experiences that you think would be useful for me to know?

Again, I want to let you know that your [resident's] medicines are not being changed based on what we have talked about. If you wish to change your [resident's] medications as a result of this discussion, we will let the care home staff know so you can discuss this with your general practitioner and any other relevant people. Please do not change your [resident's] medicines until you have talked with the appropriate healthcare professional.

Thank you very much for meeting with me. Your time is very much appreciated and your comments have been very helpful.

To finish, I am going to ask a few questions to get some basic information about you.
