# Supplementary material for: Determinants of implementing deprescribing for older adults in English care homes: a qualitative interview study
Source: BMJ Open. 2023 Nov 23;13(11):e081305. doi: 10.1136/bmjopen-2023-081305 (PMC10668129; doi:10.1136/bmjopen-2023-081305)
Supplement: Supplementary data [file bmjopen-2023-081305supp002.pdf]

STOPPING CODEBOOK

Characteristics of deprescribing	
Evidence Strength & Quality	Definition: Stakeholders' perceptions of the quality and validity of evidence supporting the belief that deprescribing will have positive outcomes for residents or will do no harm (adverse outcomes).<br><br>Inclusion Criteria: Include statements regarding awareness of evidence that deprescribing will work including the strength and quality of evidence (e.g. NICE guidelines, news articles), as well as the absence of evidence or a desire for different types of evidence.<br><br>Exclusion Criteria: Exclude or double code statements regarding the receipt of evidence as an engagement strategy to *Process, Key Stakeholders*. For example, statements regarding what evidence is needed for engagement.
Relative Advantage	Definition: Stakeholders' perception of the outcome deprescribing (i.e. better, worse, no change) versus an alternative solution (i.e. continuing medications). Split into **organisation level** (care home staff) and **individual level** (residents and families) including statements on the advantages and disadvantages of deprescribing.<br><br>Inclusion Criteria: Include statements that demonstrate the deprescribing is better (or worse/the same) than not changing medication.<br><br>Exclusion Criteria: Exclude statements that demonstrate a strong need for deprescribing and/or that the current situation is untenable and code to *Tension for Change*.
Adaptability	Definition: The degree to which deprescribing can be adapted, tailored, refined, or reinvented to meet needs at **organisation/provider** (care home) and **individual** (resident) level.<br><br>Inclusion Criteria: At **organisation level,** include statements regarding the (in)ability to adapt deprescribing to care home context e.g., perceptions on whether a different approach is needed. **Individual level** determinants may include statements on flexible, holistic and person-centered approaches.
Trialability	Definition: The ability to test deprescribing on a small scale in the care home, and to be able to reverse course (undo implementation) if warranted (i.e. restart medications).<br><br>Inclusion Criteria: At **organisation level**, include statements related to whether the care home piloted deprescribing for a short period of time (in the past or has plans to in the future) and comments about whether they believe it is (im)possible to stop medications for a short period of time. At **individual level**, include statements about resident's views on stopping medications for a short time.

Complexity	Definition: Perceived difficulty of the deprescribing. On an **organisation/provider** level, this is reflected by duration, scope, radicalness, disruptiveness, centrality, and intricacy and number of steps required to implement. On an **individual** level this is reflected by individual complexities such as severity of health condition or cognitive abilities.<br><br>Inclusion Criteria: Code statements regarding the complexity of the deprescribing process.<br><br>Exclusion Criteria: Exclude statements regarding the complexity of implementation and code to the appropriate CFIR code, e.g., difficulties related to space are coded to *Inner setting, Available Resources* and difficulties related to engaging participants in deprescribing are coded to *Process, Innovation Participants*.
Design Quality & Packaging	Definition: Perceived excellence in how the deprescribing tool, guidance or approach is bundled, presented, and assembled.<br><br>Inclusion Criteria: Include statements regarding the quality of the materials and packaging.<br><br>Exclusion Criteria: Exclude statements regarding the presence or absence of materials and code to *Available Resources*.<br>Exclude statements regarding the receipt of materials as an engagement strategy and code to *Engaging*
Cost	Definition: Costs of deprescribing and costs associated with implementing deprescribing including investment, supply, and opportunity costs.<br><br>Inclusion Criteria: Include statements related to the cost of deprescribing and its implementation. Code statements related to medication use including waste (implied cost).<br><br>Exclusion Criteria: Exclude statements related to physical space and time, and code to *Available Resources*. In a research study, exclude statements related to costs of conducting the research components (e.g., funding for research staff, participant incentives).
**Outer Setting**	
Cosmopolitanism	Definition: The degree to which the care home is networked with other external organisation i.e. primary care networks (**community/systems** level) and family carers.<br><br>Inclusion Criteria: At a **community/systems level**, include descriptions of outside group memberships and networking done outside the care home. For example, communication between the care homes with GPs and pharmacists. At an **individual level**, include statements about communication between family carer, GP and care home, also double-code to innovation participants.

	Exclusion Criteria: Exclude statements relating to information sharing or networking internally code to *Inner setting, Networks & Communication.*<br>Exclude statements relating to stakeholder role in deprescribing and code in *Process, external change agents/key stakeholders*.
External Policy & Incentives	Definition: A broad construct that includes external strategies to spread innovations including policy and regulations (governmental or other central entity), external mandates, recommendations and guidelines, pay-for-performance, collaboratives, and public or benchmark reporting.<br><br>Inclusion Criteria: Include descriptions of external performance measures from the system. For example, requirements for annual medication reviews (Quality Outcome frameworks).
**Inner Setting**	
Resident Needs & Resources	Definition: The extent to which the needs of care home residents, as well as barriers and facilitators to meet those needs, are accurately known and prioritized by the care home (**organisation/provider** level).<br><br>Inclusion Criteria: Include statements demonstrating (lack of) awareness of the needs and resources of care home residents. Analysts may be able to infer the level of awareness based on statements about: 1. Perceived need for deprescribing based on care home residents and if deprescribing will meet those needs; 2. Barriers and facilitators of care home residents to participating in deprescribing.<br><br>Exclusion Criteria: Exclude statements that demonstrate a strong need for the innovation and/or that the current situation is untenable and code to *Inner setting*, *Tension for Change*.<br>Double code statements relating to resident and family perception of need for deprescribing (**individual** level) to *Process, Innovation participants.*
Networks & Communications	Definition: The nature and quality of webs of social networks, and the nature and quality of formal and informal communications within the care home (**organisation/provider** level).<br><br>Inclusion Criteria: Include statements about general networking, communication, and relationships in the care home, such as descriptions of meetings, email groups, or other methods of keeping people connected and informed, and statements related to working relationships. Includes how care home staff know when and why medication has been changed (or not).<br><br>Exclusion Criteria: Exclude statements related to knowledge and information regarding deprescribing and code to *Access to*

	Knowledge & Information. Exclude statements related to stakeholder's (care home staff) role in implementation and code to *Process, Key Stakeholders.* Exclude descriptions of external group memberships and networking done outside the organisation and code to *Outer setting, Cosmopolitanism.*
Tension for Change	Definition: The degree to which stakeholders perceive the current situation as intolerable or needing change.<br><br>Inclusion Criteria: Include statements that (do not) demonstrate a strong need for deprescribing and/or that the current situation is untenable. For example, statements acknowledging residents are on 'too many' medication and suggest 'need' to change/deprescribe.<br><br>Exclusion Criteria: Exclude statements regarding specific needs of individuals that demonstrate a need for deprescribing, but do not necessarily represent a strong need or an untenable status quo, and code to *Inner setting, Resident Needs and Resources.*
Available Resources	Definition: The level of resources the care home (**organisation/provider** level) dedicated for making changes to the way things are done and on-going operations including physical space and time.<br><br>Inclusion Criteria: Include statements related to the presence or absence of sufficient resources specific to deprescribing that is being implemented.<br><br>Exclusion criteria: At a **community/system** level, resource changes/challenges as a result of COVID-19 double-coded as *Outer Setting, External Policy*. For example, reduced in-person medication reviews (impacting **organisation/provider** and **individual** levels).
Access to Knowledge & Information	Definition: Ease of access to digestible information and knowledge about deprescribing and how to incorporate it into the way staff provide care for residents.<br><br>Inclusion Criteria: Include statements related to care home staff access to knowledge and information regarding deprescribing (**organisation/provider**).
Compatibility	Definition: The degree of tangible fit between meaning and values attached to deprescribing by involved individuals, how those align with individuals' own norms, values, and perceived risks and needs, and how the deprescribing (including tools, guidance and approaches) fits with existing workflows and systems.<br><br>Inclusion Criteria: Include statements that demonstrate the level of compatibility deprescribing has with organizational values and work processes. Include statements that the innovation did or did not need to be adapted.<br><br>Exclusion Criteria

	Exclude or double code statements regarding the priority of deprescribing on compatibility with organizational values to *Relative Priority* e.g., if an deprescribing is not prioritized because it is not compatible with organizational values.
**Individual characteristics (staff and resident determinants)**	
Knowledge & Beliefs	Definition: Individuals' attitudes toward and value placed on the deprescribing, as well as familiarity with facts, truths, and principles related to deprescribing.<br><br>Inclusion Criteria: Include statements from care home staff (**organisation/provider**), residents and families (**individual**) about their attitudes and opinions about deprescribing. For example, statements about generational differences in accepting opinion of doctor regarding medication.<br><br>Exclusion Criteria: code statements related to stopping medication for a short time to *Intervention Characteristics, Trialability*. Double code knowledge of prescriptions (rationale, benefits) to *Inner setting>Resident needs and resources*.
Self-efficacy	Definition: Individual belief in their own knowledge, capabilities, and ability to action deprescribing.<br><br>Inclusion Criteria: Includes statements related to ability to question amount of medication on both an **organisation/provider** (care home staff) and **individual** (residents and families) level. Not only cognitive aspects (i.e. beliefs in own ability to question prescriptions) but also practical aspects (i.e. staff role restrictions).<br><br>Exclusion criteria: Double code information about staff role restrictions to *Process>Key Stakeholders* or *Outer Setting>External Policy*
**Process**	
Planning	Definition: The degree to which a scheme or method of behavior and tasks for implementing deprescribing are developed in advance, and the quality of those schemes or methods.<br><br>Inclusion Criteria: Include evidence of pre-implementation diagnostic assessments and planning (i.e. what should be done before deprescribing). For example, medication review, physical health checks. Include the perspectives of care home staff (**organisation/provider**) and residents (**individual**).
External Change Agents	Definition: Individuals who are affiliated with *outside* care home stakeholders who formally influence or facilitate deprescribing. These include GPs and pharmacists (**community/system** level).<br><br>Inclusion Criteria: Include statements related to how the external change agent (e.g. primary care networks) became engaged with

	deprescribing and what their *role* is in implementation. For example, their capabilities, motivation, and skills.<br><br>Exclusion Criteria: Note: It is important to clearly define what roles are external and internal to the organisation. If staff member *internal* to the organisation, it should be coded elsewhere (*Key stakeholders*) even though their support may overlap with what would be expected from an External Change Agent. Although family members are external to the organisation, information about role should be coded to Innovation Participants due to their position in advocating for the health of the resident/their relative.
Key Stakeholders	Definition: Care home staff role from *within* the care home (**organisation/provider** level) that are directly impacted by deprescribing.<br><br>Inclusion Criteria: Include statements related to how care home staff became engaged with deprescribing and what their role is in implementation. For example, staff perception of their role in challenging resident prescriptions, requesting reviews, initiating deprescribing discussions, reporting drug errors (professional fear of malpractice).<br><br>Exclusion Criteria: Exclude statements about general networking, communication, and relationships in the organisation, such as descriptions of meetings, email groups, or other methods of keeping people connected and informed, and statements related to team formation, quality, and functioning, and code to *Inner setting, Networks & Communications.* If statements relate to belief in capabilities rather than practical roles then code to *Individual Characteristics, Self-efficacy.*
Innovation Participants	Definition: Care home resident role in deprescribing (**individual** level). Links with family carers.<br><br>Inclusion Criteria: Include statements that capture whether or not resident needs and preferences are included in decisions around deprescribing, how residents are engaged/involved with deprescribing, and difficulties related to engaging residents in deprescribing.<br><br>Exclusion Criteria: Exclude statements demonstrating (lack of) awareness of the needs and resources of those served by the organisation and code to *Inner setting, Resident Needs & Resources> Organisation level.*
Reflecting & Evaluating	Definition: Quantitative and qualitative feedback about the progress and quality of deprescribing. Includes statements about what should happen after or be the outcome of deprescribing.

	Inclusion Criteria: Include statements that refer to the care homes (lack of) assessment of the impact and outcomes related to deprescribing. For example, how deprescribing can be done better. When participants discuss the need for monitoring following deprescribing.<br><br>Exclusion Criteria: Exclude statements that capture reflecting and evaluating that participants may do during the interview, for example, related to the success of the implementation, and code to *Individual Characteristics, Knowledge & Beliefs (about deprescribing)*.
