# Supplementary material for: Determinants of implementing deprescribing for older adults in English care homes: a qualitative interview study
Source: BMJ Open. 2023 Nov 23;13(11):e081305. doi: 10.1136/bmjopen-2023-081305 (PMC10668129; doi:10.1136/bmjopen-2023-081305)
Supplement: Author's manuscript [file bmjopen-2023-081305.draft_revisions.pdf]

BMJ Open is committed to open peer review. As part of this commitment we make the peer review history of every article we publish publicly available.

When an article is published we post the peer reviewers' comments and the authors' responses online. We also post the versions of the paper that were used during peer review. These are the versions that the peer review comments apply to.

The versions of the paper that follow are the versions that were submitted during the peer review process. They are not the versions of record or the final published versions. They should not be cited or distributed as the published version of this manuscript.

BMJ Open is an open access journal and the full, final, typeset and author-corrected version of record of the manuscript is available on our site with no access controls, subscription charges or pay-per-view fees (http://bmjopen.bmj.com).

If you have any questions on BMJ Open's open peer review process please email info.bmjopen@bmj.com

BMJ Open

DETERMINANTS OF IMPLEMENTING DEPRESCRIBING FOR OLDER ADULTS IN ENGLISH CARE HOMES: A QUALITATIVE INTERVIEW STUDY

Journal:	*BMJ Open*
Manuscript ID	bmjopen-2023-081305
Article Type:	Original research
Date Submitted by the Author:	24-Oct-2023

Complete List of Authors:	Warmoth, Krystal ; University of Hertfordshire,<br>Rees, Jessica; King's College London, Institute of Gerontology<br>Day, Jo; University of Exeter, Health and Community Sciences<br>Cockcroft, Emma; University of Exeter Medical School, Clinical Trials Unit<br>Aylward, Alex; Patient and Public Involvement Group<br>Pollock, Lucy; Somerset NHS Foundation Trust<br>Coxon, George; Classic Care (Devon) Homes Ltd<br>Craig, Trudy; Somerset Care Ltd<br>Walton, Bridget; National Institute for Health Research Applied Research Collaboration South West Peninsula<br>Stein, Ken; University of Exeter Medical School, PenTAG

Keywords:	Implementation Science, QUALITATIVE RESEARCH, Health Services for the Aged, Patient-Centered Care

SCHOLARONE™
Manuscripts

I, the Submitting Author has the right to grant and does grant on behalf of all authors of the Work (as defined in the below author licence), an exclusive licence and/or a non-exclusive licence for contributions from authors who are: i) UK Crown employees; ii) where BMJ has agreed a CC-BY licence shall apply, and/or iii) in accordance with the terms applicable for US Federal Government officers or employees acting as part of their official duties; on a worldwide, perpetual, irrevocable, royalty-free basis to BMJ Publishing Group Ltd ("BMJ") its licensees and where the relevant Journal is co-owned by BMJ to the co-owners of the Journal, to publish the Work in this journal and any other BMJ products and to exploit all rights, as set out in our licence.

The Submitting Author accepts and understands that any supply made under these terms is made by BMJ to the Submitting Author unless you are acting as an employee on behalf of your employer or a postgraduate student of an affiliated institution which is paying any applicable article publishing charge ("APC") for Open Access articles. Where the Submitting Author wishes to make the Work available on an Open Access basis (and intends to pay the relevant APC), the terms of reuse of such Open Access shall be governed by a Creative Commons licence – details of these licences and which Creative Commons licence will apply to this Work are set out in our licence referred to above.

Other than as permitted in any relevant BMJ Author's Self Archiving Policies, I confirm this Work has not been accepted for publication elsewhere, is not being considered for publication elsewhere and does not duplicate material already published. I confirm all authors consent to publication of this Work and authorise the granting of this licence.

For peer review only

DETERMINANTS OF IMPLEMENTING DEPRESCRIBING FOR OLDER ADULTS IN ENGLISH CARE HOMES: A QUALITATIVE INTERVIEW STUDY

For peer review only

Abstract

Objectives: To explore the factors that may help or hinder deprescribing practice for older people within care homes.

Design: Qualitative semi-structured interviews using framework analysis informed by the Consolidated Framework for Implementation Research.

Setting: Participants were recruited from two care home provider organisations (a smaller independently-owned organisation and a large organisation) in England.

Participants: A sample of 23 care home staff, 8 residents, 4 family members, and 1 general practitioner (GP) were associated with 15 care homes.

Results: Participants discussed their experiences and perceptions of implementing deprescribing within care homes. Major themes of (1) deprescribing as a complex process and (2) internal and external contextual factors influencing deprescribing practice (such as beliefs, abilities, and relationships) were interrelated and spanned several Consolidated Framework for Implementation Research constructs and domains. The quality of local relationships with and support from healthcare professionals were considered more crucial factors than the type of care home management structure.

Conclusions: Several influencing social and contextual factors need to be considered for implementing deprescribing for older adults in care homes. Additional training, tools, support, and opportunities need to be made available to care home staff so they can feel confident and able to question or raise concerns about medicines with prescribers. Further work is warranted to design and adopt a deprescribing approach which addresses these determinants to ensure successful implementation. ~~Deprescribing implemented safely and successfully in care homes can benefit residents.~~

For peer review only

Keywords: Polypharmacy, Medicine optimisation, Implementation science, Qualitative Study, Older adults, Homes for the Aged

Strengths and limitations of this study

- Data collection and analysis were informed by a comprehensive, well-recognised implementation science framework.
- This research adopted a strong patient and public involvement and partnership approach.
- The number of respondents who participated during the pandemic and the high demand for healthcare services should be acknowledged.
- Majority of participants were care home staff so the findings reflect mostly their perspectives and experiences.
- Methods were modified from the original protocol due to the COVID-19 pandemic.

For peer review only

Introduction

Older people living in UK care homes often experience polypharmacy, which is defined as receiving five or more concurrent medicines a day (1). In England, the 2021 National Overprescribing Review found older people to be at greater risk from polypharmacy; over half of the people over the age of 80 take eight or more medicines a day (2), many of whom live in care homes. Polypharmacy is widespread, with over 60% of residents taking five or more medicines (3), and increasing with system-wide overprescribing (2, 4). Some older adults are prescribed multiple medicines that are unlikely to improve clinical outcomes, are clinically unnecessary, or may lead to harm (5-8). One-half of care home residents are exposed to potentially inappropriate medicines (9). Care home staff, residents and family members stress the high prevalence, fears about the health and safety consequences, and burden of polypharmacy in care homes (10).

Reducing or stopping prescription medicines which may no longer be providing benefit or where the harms outweigh the benefits, known as deprescribing (11), can mitigate these harms and be safe (12-15). National Institute for Health and Care Excellence (NICE) recommends deprescribing as part of the comprehensive medication review of a person with multiple long-term conditions (16-18). While appropriate deprescribing is usually commended and may be cautiously undertaken to good effect (15), there is a lack of information about how to implement it safely and appropriately (19). Recommendations overlook the specific contextual factors and various stakeholder views of care homes (20). Deprescribing in this setting can be challenging due to the different concerns of residents, staff, clinicians, and family members and differences in care home structures.

Previous deprescribing research explored generic views towards barriers and facilitators to deprescribing (e.g., 21, 22-27) and only focused on the perspectives of a single stakeholder group, such as patients (e.g., 25, 27) or general practitioners (e.g., 22, 23, 24),

For peer review only

and rarely included crucial care home staff views (28). Different stakeholder groups from long-term care facilities prioritise different factors (29). There is a need for a better understanding of the factors influencing how deprescribing is implemented considering different stakeholders.

A recent summary of the perceptions of deprescribing in long-term care facilities from nine different countries found social influences and environmental factors were perceived as the key barriers and enablers (30). Considering this, the present study investigated the process of deprescribing and these contextual factors in English care homes, including the roles and relationships of different stakeholders, which influence it. Furthermore, implementation activities of deprescribing in care homes are typically not well-described and poorly understood (19, 31). A better understanding of how contextual factors facilitate or hinder deprescribing, informed by an implementation framework, is needed to support the translation of deprescribing recommendations into practice (32, 33).

The present paper presents the findings of the first work package of the STOPPING project (for the original protocol, see 34). The overall aim was to investigate the factors which influence deprescribing for older adults in care homes. The specific objectives of the research were to:

1. Identify the factors which influence deprescribing for older adults in care homes;
2. Explore factors influencing deprescribing across a variety of stakeholders and care home organisations; and
3. Use the Consolidated Framework for Implementation Research (CFIR) to help understand the possible determinants of implementing deprescribing for older adults in a care home setting.

For peer review only

Methods

Design

We used qualitative methods and adopted a pragmatic approach. Originally, focus groups with care home staff and healthcare professionals and in-person observations of care homes were planned but were no longer feasible due to the COVID-19 pandemic. The original methods were changed to allow for remote data collection. Individual or dyadic interviews were conducted with all participants via Microsoft® Teams, Zoom, or by telephone.

We gathered and compared perspectives from various stakeholders from two different types of care home provider organisations and used an implementation science framework to inform the data collection and analysis. The CFIR was used as an overarching framework to ensure that implementation was central to data collection and analysis (35). CFIR (2009 version) is a well-established, theoretically based implementation science framework, comprised of 39 constructs divided into five domains (35). CFIR focuses on identifying and understanding constructs that can shape the implementation and the routinisation of health services (36), making it appropriate for care homes.

Setting

The two care home providers were selected for this research as they represent contrasting models of care home provision. That is, one is a smaller independently-owned organisation consisting of two residential care home sites and a large organisation with more than 25 care homes in several locations across England. They were also selected as they mainly care for older adults (over 65 years old). Care home sites included in this study were selected in partnership between the research team and senior care home staff.

For peer review only

Participants

Recruitment and data collection occurred from December 2021 to May 2022. Table 1 for participant details and Table 2 for the care home details. Care home and other healthcare staff were eligible for the study if they were currently working directly with older adults with polypharmacy and/or in the care home setting and could converse in English without an interpreter or professional assistance. Participants were purposefully recruited from distinct types of care home providers using established networks of care home organisations. Care home staff were selected and approached by the regional managers or directors of the care home organisations, who were known to the researchers.

Care home staff approached residents and their family members about taking part in the study. The eligibility criteria for care home residents taking part in the research were being a resident of the participating care home, aged 65 and over, taking multiple medications or having experience of polypharmacy, ability to converse in English without an interpreter or professional assistance, and having an absence of serious cognitive impairment, as identified by the care home staff or healthcare profession, and have capacity to consent to participation. Care home residents' family members or friends were eligible if their resident was taking multiple medications at the care home participating in the study and ability to converse in English without an interpreter or professional assistance. Residents with severe cognitive impairment which inhibits consent were excluded, but their family members and carers were included, as this group are particularly at risk of overall drug burden and often benefit from deprescribing interventions.

Data collection

Demographic data were collected from all participants following the interview with a brief survey. Semi-structured interviews were conducted with residents, their family members, care home staff, and healthcare professionals about their experiences and beliefs to

For peer review only

identify the factors influencing deprescribing and the current deprescribing practice in different care home settings. Interview topic guides were informed by CFIR constructs identified in previous literature (e.g., 21, 25-27). See supplementary files for the interview guides. Interviews were conducted by a qualified female researcher with more than 10 years experience (BLINDED FOR REVIEW) and audio-recorded and professionally transcribed verbatim for analysis. Reflective notes were taken to supplement the transcripts. NVivo 12 was used to manage the data.

Qualitative data collection was conducted until data saturation occurred, meaning that no new ideas are being generated. The sample size was determined using previous literature on qualitative methods and using purposeful sampling. We estimated the minimum sample size of 24 was needed, based on work that found data saturation and variability to be present as early as six interviews (37).

Data analysis

A Framework Analysis approach (38) was employed for data analysis. A CFIR-informed codebook was adapted to address the research question and to consider distinct levels of analysis (individual, organisational and community); see supplementary files. Transcripts were deductively coded within the domains and constructs of CFIR by a single researcher (BLINDED FOR REVIEW). Then, researchers (BLINDED FOR REVIEW) developed the major themes across the domains inductively. Discussions with patient and public involvement (PPI) representatives helped to refine the major themes and the implications of the research.

Patient and public involvement

This research adopted a strong patient and public involvement and partnership approach with residents, staff, and family carers. Before data collection, a PPI workshop was

For peer review only

held with care home residents and staff at one care home. This workshop informed the production of study materials and ensured they were appropriate for and understandable by care home residents and staff. A pilot interview with an independent member of a local PPI group was conducted, and as a result, opportunities for breaks were included and minor changes to the wording of the topic guides were made.

Researchers (BLINDED FOR REVIEW) and a PPI representative (BLINDED FOR REVIEW) met regularly to discuss the analysis plan, review and refine the codebook, develop the themes, and interpret the findings.

A final PPI workshop was held to discuss the findings and refine the dissemination strategy with a group of family carers and care home workers. Two researchers and the PPI representative presented the findings and facilitated group discussions about the interpretation of the findings and dissemination. Attendees stated how they shared similar experiences with participants and the varied procedures in care homes.

Results

In total, 36 interviews were conducted with 23 care home staff, 8 residents, 4 family members, and 1 general practitioner (GP) who were associated with 15 care homes. Interviews lasted on average approximately 41 minutes (ranging from 16 to 94 minutes). Two major themes were developed: 1) *Deprescribing as a complex process* and 2) *Internal and external contextual factors* influencing deprescribing in care homes. These themes were interrelated and spanned several CFIR constructs and domains. See Table 3 for the major themes, CFIR determinants, and supporting quotes.

Deprescribing as a complex process

This theme related to the activities and strategies described to implement deprescribing in a care home setting, relating to the CFIR domain of process. Participants

For peer review only - http://bmjopen.bmj.com/site/about/guidelines.xhtml

discussed deprescribing as a complex process with multiple steps. It included preparation and planning, involvement of multiple people with different roles, and ongoing monitoring and evaluation.

Preparation and planning deprescribing

Conversations about deprescribing could be initiated by a regular medication review, an observed change in the resident, or the resident's preference. Before stopping or reducing medications, care home residents and staff discussed that "*health checks*'' and comprehensive medication reviews should be conducted. Care home staff and the GP expressed how, during a medicine review, there was a need to fully understand the resident's medications and the rationale for each one's use. If a decision was made to deprescribe then the care home needed to know how to do it (i.e. tapering over time or stopping straightaway). Care home staff discussed supporting resident involvement and its difficulties with some residents (e.g., cognitive impairment). They considered that it was important to explain any changes and review how the resident was feeling on certain medications, before stopping or reducing medications.

Engaging multiple people

For deprescribing to happen, all participants discussed how it involved the engagement of various individuals, internal and external to the care homes. Each had a specific role and expertise, which was highlighted by participants. Care home staff, residents and their families expressed how care home staff are residents' advocates and/or intermediaries and often ''*know them best''* (i.e. the resident's individual needs and resources); whereas GPs made the ultimate decisions about if and how deprescribing should happen. A resident discussed the distinct roles and how they were ''*interdependent because they depend on each other.*'' The involvement of these different people and their respective knowledge and beliefs could help and hinder deprescribing. For example, family members

For peer review only

were discussed by care home staff as either encouraging or objecting to the resident's medicines being deprescribed.

The role of a pharmacist in the deprescribing process was more ambiguous. Some residents and care home staff thought the role of a pharmacist was only dispensing medication and, hence, were not involved in any aspect of deprescribing or unsure how they could help. Conversely, if a care home had experienced additional support from pharmacists, such as leading medicine reviews or as a resource for questions about medicines, they were more likely to report how a pharmacist's input was valuable to deprescribing. Pharmacists were considered experts in medicines, but the ultimate decision is made by the GP unless the resident had secondary care where another clinician makes prescribing decisions. Despite the knowledge and expertise of care home staff about the resident and the pharmacist about medicines, all participants (especially, residents) expressed how deprescribing decisions would be done by the GP. Occasionally, other healthcare professionals (e.g., the mental health team, geriatricians, or specific condition nurses) were mentioned but they were often described as being consulted by the GP to make their decisions.

Monitoring and evaluation

Another key part of the deprescribing process that participants discussed was the monitoring and evaluation after medication changes. This was a consistent theme across all participant groups but especially the care home staff as they were the ones observing residents following medication changes. Care home staff emphasised how important it was for them to know about medications to make relevant observations regarding any benefits or side effects. The types of observations required depend on which medications were changed (e.g., blood pressure, behaviour, alertness, and appetite). The timings and workload required for feedback also varied depending on the complexity of the resident's health and the types of medication changes. Care home staff and the GP discussed how the effects of stopping or

For peer review only

reducing certain medications may take longer than others, thus impacting the monitoring, feedback, and review process. This monitoring was described as actively ongoing to optimise medications for residents and included the possibility of re-starting medications.

Internal and external contextual factors

This theme concerns the contextual factors, including beliefs, abilities, and relationships, reported to influence deprescribing in a care home setting. These CFIR determinants are related to the characteristics of staff and residents (views about deprescribing benefits, ability to question and contribute) as well as the relationships and communication between care homes and the healthcare community (e.g., GPs, pharmacists, and hospitals).

Views about deprescribing and its benefits

Most participants, especially care home staff, reported having mostly positive views and experiences of deprescribing. Often, staff and residents articulated that if medicines were not needed or did not improve the resident's well-being then they would be happy to support deprescribing. Some residents expressed a few concerns because some medicines were perceived as indispensable. Residents discussed concerns about the consequences if these were deprescribed and how some medicines were necessary or too important (for example, antipsychotics for schizophrenia). Care home staff reported the various potential benefits to resident health, quality of life, costs, time, and safety. All participants thought that a deprescribing process should be individualised to the resident and, therefore, adaptable, pilotable, and reversible. These beliefs and attitudes of individuals seem to instil a dynamic, tailored, responsive deprescribing culture in the care home.

Ability to question and contribute

A key characteristic discussed by care home staff and residents was the capacity and confidence to question or raise concerns about medicines with GPs or other prescribers.

For peer review only

However, most care home staff and residents felt that they would default to GP advice. Care home staff stated how they did not have the training to question prescribing decisions, but they conveyed that questioning medicines was important to advocate for residents. For those staff who did feel confident to question or raise concerns, the expectation was that they would be listened to by the doctor. Care home staff recognised their knowledge about residents and wanted it to be valued in deprescribing conversations and decisions.

Relationships and communication

Care home staff discussed how mutual trust and respect between care homes and healthcare staff enabled collaboration and good working relationships. The collaboration between GPs and care homes was considered a crucial determinant for deprescribing, despite the support of other healthcare professionals (e.g., pharmacists and other specialists). Supporting the deprescribing process and this collaboration was a good working relationship and information sharing between primary care and the care home. Care homes relied on professionals to review medications, deprescribe, and provide information about monitoring following changes; while the GP relied on the feedback provided by the care home on resident health. The exchanges and information sharing between care home staff and GPs (and other healthcare professionals) determined the access to knowledge and evidence to facilitate deprescribing. This communication was considered an essential component of the deprescribing process. For example, care home staff, the GP and residents discussed how deprescribing could be hindered if medication changes were not communicated to the care home. Care home staff also discussed their role in keeping family members informed, which was described as more challenging when they were unable to visit homes during the pandemic.

Notably, the distinct types of care home providers (independently owned and part of a larger organisation) did not seem a crucial factor influencing deprescribing. More critical

For peer review only

were the working relationships with GP practices and support at individual care homes (such as regular medication reviews or access to pharmacists). The local quality of the relationships and how they worked with healthcare professionals could vary greatly among the providers and individual care homes.

Discussion

The overall aim of this qualitative study was to identify factors which influence deprescribing in English care home settings. The major themes found related to how deprescribing was a complex process, with multiple steps and the involvement of multiple people with distinct roles, and internal and external contextual factors, concerning the characteristics of staff and residents and communication and relationships with the wider healthcare community. Local context and available support were found to be crucial factors across types of care home providers.

The findings add to previous deprescribing research, which described the barriers and enablers (e.g., 21, 22-27, 29); this study described in detail the process of deprescribing and the contextual factors which influence it in the English care home setting. It also considered the various roles and relationships of these different individuals involved in deprescribing. The present study found that how these different people worked together was an important determinant of deprescribing. Participants discussed and stressed how important collaboration and working relationships between care homes and healthcare providers (e.g., GPs and other prescribers) more than the organisational structure of the care home provider. This finding suggests that these roles and relationships need to be addressed in any successful deprescribing approach in care homes.

It must be recognised that there are power differentials and a ‘hierarchy’ (with GPs holding the decision-making power) in these relationships, and the management of care home residents’ medicines has to take into consideration who has decision-making power (30).

For peer review only

Hierarchies and power differentials can affect whose voice is heard; it is well-documented that when power differentials between health and social care settings are present healthcare priorities invariably dominate (39). Fostering collective purpose and identity across sectors could ensure communication and collaboration are not negatively affected (40). Care home residents, family members, care home staff and relevant healthcare professionals should be involved in the deprescribing process (if possible and appropriate) and their respective knowledge recognised and valued for shared decision-making. Additional training, tools, support, and opportunities may need to be made available to care home staff so they can feel confident and able to question or raise concerns when they think that something isn't quite right with the medication. Known enablers of psychological safety and patient safety culture in healthcare teams (such as, professional responsibility, open communication, peer support change-oriented leadership, and learning orientation) could be implemented (41, 42). Future research could examine these separate roles and working relationships as well as resources to support them in greater detail to determine what activities and strategies encourage appropriate deprescribing.

Previous research has recommended nurse champions deprescribing in long-term care facilities (30), but UK care homes do not always have access to qualified nurses. A recent trial introduced pharmacists to lead deprescribing in UK care homes (43), but similar to this study's findings, the role of the pharmacist was not always understood by care home staff. Any initiative will need to educate not only those individuals selected to champion deprescribing but also the rest of those involved so there is a shared understanding of each other's role and contribution. The involvement of multiple individuals and organisations also raises issues related to legal concerns, higher workloads, and duty of care (21). Successful integrated working requires trust between health professionals and a clear understanding of

For peer review only

responsibilities (44). Further work may need to explore how these are established and negotiated in the care home-primary care working relationships.

The findings support previous work and policy promoting the involvement of multidisciplinary teams (30) and collaboration across care settings (45) to facilitate and implement deprescribing safely and efficiently. Recent policy changes in England set out standards for primary care services to support the delivery of the 'Enhanced Health in Care Homes' framework (46). A major component of enhanced primary care support includes routine structured medication reviews. This mandated support from primary care is not only an impetus for medicine reviews and potential deprescribing in care homes but also provides regular opportunities for planning, decision-making, and reflecting on their practice. But, evidence suggests that the adoption of the Enhanced Health in Care Homes Framework is not universal or uniform (47). Accordingly, not all care homes will have access to the same support for deprescribing so approaches may need to be tailored to the availability of local resources and, as this study found, the resources and relationships for individual care homes were key factors influencing deprescribing. The findings contribute to the medicine optimisation national priority (18, 48, 49) and NICE recommendations for deprescribing in care homes (17, 18). With an increasing ageing population and greater demand for care in the community, medicine optimisation will continue to be a priority for healthcare services.

Strengths and limitations

A strength of this work is the use of a comprehensive, well-recognised implementation science framework, CFIR, to investigate how to implement deprescribing in real-world settings (20, 50), specifically in care homes (51). The project demonstrates how CFIR can be used to identify the factors influencing deprescribing into practice (32, 33). The present study findings denoted several domains and constructs of CFIR and the relationships between these determinants. Using this framework can identify crucial determinants, address

For peer review only

barriers to deprescribing and help ensure that interventions are effective and sustainable (33, 52). Another strength of the study was the number of respondents that participated in the context and conditions of the pandemic and the high demand for healthcare services where care staff have been so overwhelmed by pressures. Finally, this research adopted a strong patient and public involvement and partnership approach which contributed greatly to the delivery and success of the study.

Some limitations of the study must be acknowledged. Due to the COVID-19 pandemic, care home observations and focus groups were not conducted, which had been originally planned (34); this could have provided a better understanding of everyday practices and insight into experiences than interviews alone. Future work could adopt an ethnographic approach to explore the culture of deprescribing in care homes and observe deprescribing conversations and behaviours. By only conducting interviews, we had to exclude participants who are unable to converse in English and show signs of severe cognitive impairment. These individuals may have different experiences and perspectives. Interviews were with family and friends of these older adults to try to capture these experiences and views. Other limitations of the study were that two-thirds of the sample were care home staff (especially, those in senior positions) and most of the participants were female and white so the findings reflect mostly their perspectives and experiences. Moreover, there may be bias in the sample as regional managers and directors were involved in the selection of participants. Participants were informed that their responses would be kept confidential, and their employment or care (or family member's care) would not be affected by their responses; they did provide both positive and negative experiences. There was only one prescriber interviewed, but as we were principally interested in how deprescribing would work in a care home setting, the recruitment of care home staff, residents and families were prioritised. Most previous

For peer review only

research has studied the views of prescribers (e.g., 22, 23, 24) and rarely included the views of care home staff.

From this study sample, a difference in views between provider types was not noted but it does not mean there one is not present. Further research could explore the views of more providers and care homes to explore possible differences. Involving care homes in research is challenging and there are several barriers to research participation (53). The COVID-19 pandemic has shown how crucial care homes and their staff are in the care of the frailest and vulnerable. The skills, dedication, and compassion of care home staff not only shape care but can greatly contribute to research.

Conclusion

For deprescribing to be successfully undertaken in care homes, several influencing factors (such as individuals' beliefs and abilities, relationships, and communication) need to be considered and the process itself of deprescribing (including preparation, engaging stakeholders and monitoring). Further work is warranted to design and implement a deprescribing approach for care homes that can be achieved within current structures and resources. ~~Deprescribing implemented safely and successfully in care homes benefits residents~~.

For peer review only

References

1. Masnoon N, Shakib S, Kalisch-Ellett L, Caughey GE. What is polypharmacy? A systematic review of definitions. BMC Geriatrics. 2017;17:230.

2. Department of Health and Social Care. Good for you, good for us, good for everybody: A plan to reduce overprescribing to make patient care better and safer, support the NHS, and reduce carbon emissions. 2021.

3. Izza MAD, Lunt E, Gordon AL, Gladman JRF, Armstrong S, Logan P. Polypharmacy, benzodiazepines, and antidepressants, but not antipsychotics, are associated with increased falls risk in UK care home residents: a prospective multi-centre study. European Geriatric Medicine. 2020;11(6):1043-50.

4. Duerden M, Avery T, Payne R. Polypharmacy and medicines optimisation: making it safe and sound. London: The King's Fund; 2013.

5. Kongkaew C, Noyce PR, Ashcroft DM. Hospital admissions associated with adverse drug reactions: a systematic review of prospective observational studies. Annals of Pharmacotherapy. 2008;42(7-8):1017-25.

6. Jyrkka J, Enlund H, Korhonen MJ, Sulkava R, Hartikainen S. Polypharmacy status as an indicator of mortality in an elderly population. Drugs Aging. 2009;26(12):1039-48.

7. Gnjidic D, Hilmer SN, Blyth FM, Naganathan V, Waite L, Seibel MJ, et al. Polypharmacy cutoff and outcomes: five or more medicines were used to identify community-dwelling older men at risk of different adverse outcomes. Journal of clinical epidemiology. 2012;65(9):989-95.

8. Turner JP, Jamsen KM, Shakib S, Singhal N, Prowse R, Bell JS. Polypharmacy cut-points in older people with cancer: how many medications are too many? Supportive care in cancer : official journal of the Multinational Association of Supportive Care in Cancer. 2016;24(4):1831-40.

For peer review only

9. Morin L, Laroche M-L, Texier G, Johnell K. Prevalence of potentially inappropriate medication use in older adults living in nursing homes: a systematic review. Journal of the American Medical Directors Association. 2016;17(9):e861-2. e9.

10. Gnjidic D, Tinetti M, Allore HG. Assessing medication burden and polypharmacy: finding the perfect measure. Expert review of clinical pharmacology. 2017;10(4):345-7.

11. Reeve E, Gnjidic D, Long J, Hilmer S. A systematic review of the emerging definition of ‘deprescribing’ with network analysis: implications for future research and clinical practice. British Journal of Clinical Pharmacology. 2015;80(6):1254-68.

12. Anderson LJ, Schnipper JL, Nuckols TK, Shane R, Sarkisian C, Le MM, et al. A systematic overview of systematic reviews evaluating interventions addressing polypharmacy. American Journal of Health-System Pharmacy. 2019;76(21):1777-87.

13. Iyer S, Naganathan V, McLachlan AJ, Le Conteur DG. Medication Withdrawal Trials in People Aged 65 Years and Older. Drugs & Aging. 2008;25(12):1021-31.

14. Kua C-H, Mak VSL, Huey Lee SW. Health Outcomes of Deprescribing Interventions Among Older Residents in Nursing Homes: A Systematic Review and Meta-analysis. Journal of the American Medical Directors Association. 2019;20(3):362-72.e11.

15. Cooper JA, Cadogan CA, Patterson SM, Kerse N, Bradley MC, Ryan C, et al. Interventions to improve the appropriate use of polypharmacy in older people: a Cochrane systematic review. BMJ Open. 2015;5(12).

16. National Institute for Health and Care Excellence (NICE). Multimorbidity: clinical assessment and management (NG56). 2016.

17. National Institute for Health and Care Excellence (NICE). Medicines optimisation: the safe and effective use of medicines to enable the best possible outcomes (NG5). 2015.

For peer review only

18. National Institute for Health and Care Excellence (NICE). 2017. Available from: https://www.nice.org.uk/advice/ktt18/resources/multimorbidity-and-polypharmacy-pdf-58757959453381.

19. Wang J, Shen JY, Conwell Y, Podsiadly EJ, Caprio TV, Nathan K, et al. Implementation considerations of deprescribing interventions: A scoping review. J Intern Med. 2022.

20. Reeve E, Thompson W, Farrell B. Deprescribing: A narrative review of the evidence and practical recommendations for recognizing opportunities and taking action. European journal of internal medicine. 2017;38:3-11.

21. Palagyi A, Keay L, Harper J, Potter J, Lindley RI. Barricades and brickwalls–a qualitative study exploring perceptions of medication use and deprescribing in long-term care. BMC geriatrics. 2016;16(1):15.

22. Schuling J, Gebben H, Veehof LJG, Haaijer-Ruskamp FM. Deprescribing medication in very elderly patients with multimorbidity: the view of Dutch GPs. A qualitative study. BMC family practice. 2012;13(1):56.

23. Moen J, Norrgård S, Antonov K, Nilsson JLG, Ring L. GPs' perceptions of multiple-medicine use in older patients. Journal of evaluation in clinical practice. 2010;16(1):69-75.

24. Anthierens S, Tansens A, Petrovic M, Christiaens T. Qualitative insights into general practitioners views on polypharmacy. BMC family practice. 2010;11(1):65.

25. Linsky A, Simon SR, Bokhour B. Patient perceptions of proactive medication discontinuation. Patient Education and Counseling. 2015;98(2):220-5.

26. Linsky A, Simon SR, Marcello TB, Bokhour B. Clinical provider perceptions of proactive medication discontinuation. Am J Manag Care. 2015;21(4):277-83.

For peer review only

27. Kalogianis MJ, Wimmer BC, Turner JP, Tan ECK, Emery T, Robson L, et al. Are residents of aged care facilities willing to have their medications deprescribed? Research in Social and Administrative Pharmacy. 2016;12(5):784-8.

28. Lo SY, Reeve E, Page AT, Zaidi STR, Hilmer SN, Etherton-Beer C, et al. Attitudes to Drug Use in Residential Aged Care Facilities: A Cross-Sectional Survey of Nurses and Care Staff. Drugs & Aging. 2021;38(8):697-711.

29. Turner JP, Edwards S, Stanners M, Shakib S, Bell JS. What factors are important for deprescribing in Australian long-term care facilities? Perspectives of residents and health professionals. BMJ Open. 2016;6(3):e009781.

30. Heinrich CH, Hurley E, McCarthy S, McHugh S, Donovan MD. Barriers and enablers to deprescribing in long-term care facilities: a 'best-fit' framework synthesis of the qualitative evidence. Age and Ageing. 2022;51(1).

31. Page AT, Clifford RM, Potter K, Schwartz D, Etherton-Beer CD. The feasibility and effect of deprescribing in older adults on mortality and health: a systematic review and meta-analysis. British Journal of Clinical Pharmacology. 2016;82(3):583-623.

32. Ailabouni NJ, Reeve E, Helfrich CD, Hilmer SN, Wagenaar BH. Leveraging implementation science to increase the translation of deprescribing evidence into practice. Research in Social and Administrative Pharmacy. 2022;18(3):2550-5.

33. Ronquillo C, Day J, Warmoth K, Britten N, Stein K, Lang I. An Implementation Science Perspective on Deprescribing. Public Policy & Aging Report. 2018;28(4):134-9.

34. Warmoth K, Day J, Cockcroft E, Reed DN, Pollock L, Coxon G, et al. Understanding stakeholders' perspectives on implementing deprescribing for older people living in long-term residential care homes: the STOPPING study protocol. Implementation Science Communications. 2020;1(1).

For peer review only

35. Damschroder LJ, Aron DC, Keith RE, Kirsh SR, Alexander JA, Lowery JC. Fostering implementation of health services research findings into practice: a consolidated framework for advancing implementation science. Implementation science. 2009;4(1):50.

36. Greenhalgh T, Robert G, Macfarlane F, Bate P, Kyriakidou O. Diffusion of innovations in service organizations: systematic review and recommendations. The Milbank Quarterly. 2004;82(4):581-629.

37. Guest G, Bunce A, Johnson L. How Many Interviews Are Enough? An Experiment with Data Saturation and Variability. Field Methods. 2006;18(1):59–82

38. Ritchie J, Lewis J, McNaughton Nicholls C, Ormston R. Qualitative research practice: A guide for social science students and researchers. 2nd ed. London: SAGE 2014

39. Gordon AL, Spilsbury K, Achterberg WP, Adams R, Jones L, Goodman C. From Warkworth House to the 21st century care homes: progress marked by persistent challenges. Age and Ageing. 2022;51(7).

40. Amador S, Goodman C, Mathie E, Nicholson C. Evaluation of an Organisational Intervention to Promote Integrated Working between Health Services and Care Homes in the Delivery of End-of-Life Care for People with Dementia: Understanding the Change Process Using a Social Identity Approach. Int J Integr Care. 2016;16(2):14.

41. O'donovan R, McAuliffe E. A systematic review of factors that enable psychological safety in healthcare teams. International Journal for Quality in Health Care. 2020;32(4):240-50.

42. Battard J. Nonpunitive response to errors fosters a just culture. Nursing Management. 2017;48(1):53-5.

43. Birt L, Dalgarno L, Wright DJ, Alharthi M, Inch J, Spargo M, et al. Process evaluation for the Care Homes Independent Pharmacist Prescriber Study (CHIPPS). BMC Health Services Research. 2021;21(1).

For peer review only

44. Davies SL, Goodman C, Bunn F, Victor C, Dickinson A, Iliffe S, et al. A systematic review of integrated working between care homes and health care services. BMC Health Services Research. 2011;11(1):320.

45. Conklin J, Farrell B, Suleman S. Implementing deprescribing guidelines into frontline practice: Barriers and facilitators. Research in Social and Administrative Pharmacy. 2019;15(6):796-800.

46. NHS England and NHS Improvement. The Framework for Enhanced Health in Care Homes;. In: England N, editor. London, UK2020.

47. Warmoth K, Goodman C. Models of Care and Relationships with Care Homes: Cross-Sectional Survey of English General Practices. International Journal of Environmental Research and Public Health. 2022;19(22):14774.

48. Naylor C, Imison C, Addicott R, Buck D, Goodwin N, Harrison T, et al. Transforming our helath care system: Ten priorities for commissioners. The King's Fund; 2015.

49. NHS England. Regional Medicines Optimisation Committee 2017 [Available from: https://www.england.nhs.uk/publication/regional-mediciens-optimisation-committee-operating-guidance-and-recruitment-information/.

50. Sawan M, Reeve E, Turner J, Todd A, Steinman MA, Petrovic M, et al. A systems approach to identifying the challenges of implementing deprescribing in older adults across different health-care settings and countries: a narrative review. Expert Review of Clinical Pharmacology. 2020;13(3):233-45.

51. Alldred DP, Kennedy M, Hughes C, Chen TF, Miller P. Interventions to optimise prescribing for older people in care homes. Cochrane Database of Systematic Reviews. 2016(2).

For peer review only

52. Gnjidic D, Le Couteur DG, Hilmer SN. Discontinuing drug treatments. BMJ. 2014: 349.

53. Law E, Ashworth R. Facilitators and Barriers to Research Participation in Care Homes: Thematic Analysis of Interviews with Researchers, Staff, Residents and Residents' Families. Journal of Long-Term Care. 2022;0(2022):49.

For peer review only

Table 1. Participant characteristics (n= 36)

	n (%)	M (SD)
**Care home staff and health care professionals (n=24)**		
Age		44.65 (12.04)
Gender		
Female	21 (87.50)	
Male	3 (12.50)	
Ethnic group		
White	23 (95.80)	
Asian	1 (4.20)	
Education		
General Certificate of Education	3 (12.50)	
Undergraduate degree	5 (20.8 0)	
National vocational qualification	14 (58.30)	
Postgraduate degree	2 (8.30)	
Employment duration (in months)		47.83 (59.27)
Key responsibilities and duties		
Assistance with daily tasks in care home	14 (58.33)	
Administering medicines	17 (70.83)	
Providing company and assistance in leisure activities	5 (20.83)	
Developing care plans	20 (83.33)	
Planning/overseeing work of other staff members	21 (87.50)	
Prescribing and medicine review	16 (66.67)	
Other	14 (58.33)	
Hours worked per week		
Less than 32 hours	3 (12.50)	
More than 32 hours	21 (87.50)	
**Family carers (n=4)**		
Age		64.75 (1.26)
Gender		
Female	3 (75.00)	
Male	1 (25.00)	
Ethnic group - White	4 (100.00)	
~~Education~~		
~~No formal~~	~~1 (25.00)~~	
~~General Certificate of Education~~	~~1 (25.00)~~	
~~Undergraduate degree~~	~~1 (25.00)~~	
~~National vocational qualification~~	~~1 (25.00)~~	
Relationship to resident		
Son	1 (25.00)	
Daughter	2 (50.00)	
Sibling	1 (25.00)	

For peer review only

**Residents (n=8)**		
Age		88.75 (5.06)
Gender		
Female	6 (75.00)	
Male	2 (25.00)	
Ethnic group - White	8 (100.00)	
Education		
No formal	3 (37.50)	
General Certificate of Education	3 (37.50)	
National vocational qualification	1 (12.50)	
Apprenticeship	1 (12.50)	
Perceived health status		3.50 (0.93)
1. Excellent	1 (12.50)	
2. Very good	0	
3. Good	3 (37.50)	
4. Fair	3 (37.50)	
5. Poor	1 (12.50)	
Number medicines daily		7.71 (3.99)
Number of conditions		6.40 (2.30)

Note. One participant could not remember the number of medicines they took daily so they were not included in the analysis for that item.

For peer review only

Table 2. Care home characteristics

	Number of participants	Number of beds	Occupancy (%)	Total number staff	Dementia speciality	Nursing care
**Large care home provider**						
Care home 1	2 residents, 1 staff	60	88	85	Yes	No
Care home 2	2 family	90	89	140	Yes	Yes
Care home 3	2 staff,<br>1 family	55	100	75	Yes	Yes
Care home 4	2 staff	34	75	36	No	No
Care home 5	1 staff,<br>1 resident,<br>1 family	54	81	57	No	No
Care home 6	1 staff,<br>1 resident	50	76	32	Yes	No
Care home 7	1 staff	68	83	74	Yes	No
Care home 8	2 staff	46	85	62	Yes	No
Care home 9	1 staff	44	68	36	Yes	No
Care home 10	3 staff,<br>2 residents	37	91	54	Yes	No
Care home 11	1 staff,<br>1 resident	40	95	49	No	No
Care home 12	2 staff	38	60	42	No	No
Care home 13	1 staff	39	79	50	Yes	No
**Small independent owned provider**						
Care home 14	3 staff,<br>1 resident	17	100	25	Yes	No
Care home 15	2 staff	19	100	26	Yes	No

For peer review only

Table 3. Summary of factors influencing the implementation of deprescribing in care homes from interviews with residents, family carers, care home staff and healthcare professionals

Major themes	CFIR domains	CFIR constructs	Definitions	Supporting quotes
Deprescribing as a process	Process	Planning	Degree to which tasks or behaviours for implementing deprescribing are developed in advance	"I think there'd be a bit of a process involved. I think probably the family members and staff, the people that know the individuals well, or better than anybody else, they'd have to have an input for their opinion. And then the person that prescribes the medicines, would have to review it and decide what they think for the best." (Care home 14, care assistant)<br><br>"I think we need to know what it is they're taking, and why they're taking it. We need to know what the adverse effect would be if they stopped taking it, and whether or not they need to be weaned off it, like less and less dose rather than just stopping it." (Care home 5, Deputy Manager)
	Engaging	External change agents	Individuals who are affiliated with organisations/services outside the care home who formally influence or facilitate deprescribing.	" When somebody becomes unwell, we always like to have a home visit, just to be able to say, be able to tell the families, well, with the GP has seen them. Because it is sometimes hard to get across over the phone, if somebody is unwell. But, ultimately, yeah, the GP, it is in their hands and we can have our opinion, but if the GP doesn't want to change any medication, then that's down to them." (Care home 7, Supervisor)<br><br>"The pharmacist poking his nose in may be good, or it may not be, I don't know." (Care home 14, resident)
		Key stakeholders	Care home staff roles that influence deprescribing	"The only thing we can do is refer it back to the specialists, because, like I said, we can't stop medication ourselves in any capacity. So all we can do is be like the middleman between the

For peer review only

				residents, and the doctors." (Care home 8, Supervisor & medication lead)<br><br>"So I think they're essential for coordinating everyone, really, and actually they're better at doing that than the GP will ever be." (Care home 8, GP)
		Innovation participants - Resident	Resident role in deprescribing (incl. how needs and preferences and included in decisions, and engagement and involvement examples/challenges)	"The resident themselves; they should have an input if they have capacity to do it, if they're able to retain the information about their medication." (Care home 12, Deputy manager)<br><br>"I don't get involved at all...because I wouldn't know what they were talking about. But if it was like we are going to change this one to this one, I'd say how would it affect me, and they'd say it won't and I would accept it." (Care home 14, resident)
		Innovation participants -Family carer	Family carer role in deprescribing (incl. how needs and preferences and included in decisions, and engagement and involvement examples/challenges)	"And if they lack capacity, even if they don't lack capacity, then their family, close family, next to kin, power of attorney, we would ask and consult them as well, for anything that changes with any of their care." (Care home 6, Deputy manager and medication lead)<br><br>"They could be biased, and not have a clear view about why we are trying to stop a medication. They might not see both sides of the story, and they'd be worried that it's being done for the wrong reasons. But we do like to keep families involved." (Care home 9, Deputy manager)
	Reflecting and evaluating		Feedback about the progress and quality of deprescribing and	"I think it is the monitoring the information, and collecting all that information. And I know it sounds like a big thing, but I think without that information, I don't think... Because you can't just

For peer review only

			monitoring following deprescribing	stop medication without monitoring, can you?" (Care home 14, Deputy manager)<br><br>"Well, I suppose a final review, at some point, so that's predetermined and there should be a point where it's agreed that there should be a review of how it was effective, but whether it's worked, I suppose." (Care home 2, family carer)
Internal and external contextual factors	Individual characteristics	Knowledge and beliefs about deprescribing-Resident	Resident attitudes and opinion towards deprescribing.	"I think I would have concerns, certainly, with some medication you have to be careful when you come off with the after-effects. And it has to be done in carefully? I wouldn't just accept everything, you have an understanding why." (Care home 1, resident)<br><br>"I know the important one shouldn't be stopped." (Care home 6, resident)
		Knowledge and beliefs about deprescribing- Care home staff	Care home staff attitudes and opinion towards deprescribing.	"Well, sometimes if you've been on a medication for so long, a lot of our residents have been on their medication for 20-odd years because, like I said, they're in their 90s. Is it still working? Is it doing in its job? Do they - let's review it and see. At 90 - in your 90s, do you need to be trying to reduce your cholesterol, really?" (Care home 9, Deputy manager)<br><br>"And they don't necessarily need it, and they come up with it, and we've trialled them coming off of it and they're absolutely fine. And it's nicer for them not being pumped full of medication, rattling around daily. If it's not needed, and they've come here to live out the rest of their life, who really wants to be taking lots of medication every day?" (Care home 12, Supervisor)

For peer review only

		Self-efficacy – Resident	Resident belief in their own knowledge, capabilities and ability to action deprescribing.	"Well, if the medical professional thought it was all right, I would just take their word for it...I think we would take the advice of the doctor." (Care home 1, resident)<br><br>"And they have so much belief in the doctor and what the doctor says, so if it's not the doctor that's saying these things, I just wonder what sort of a response you would get." (Care home 2, family carer)
		Self-efficacy- Care home staff	Care home staff belief in their own knowledge, capabilities and ability to action deprescribing.	"We would never make those judgements on our own, and we have our opinions if we think something's not working or something needs to be in place, something, a new prescription, so then we obviously work closely with the GPs to discuss that." (Care home 7, Supervisor)<br><br>"I think we're able to certainly speak up and challenge. I don't think we'd start questioning and going, they shouldn't be on this, but we would certainly raise concerns and making sure that people are having regular medication reviews, so that they're not on anything unnecessarily." (Care home 4, Deputy manager)
	Outer setting	Cosmopolitanism	The degree to which the care home is networked with other external organisations	"Of course, it's interdependent, because they depend on each other...it depends on the advice of the carer giving to the doctors about what's happening to a patient. And then the doctors summing it up and advising that they should have a certain medication specifically for that patient, or that resident." (Care home 6, resident)<br><br>"The pharmacist comes - at the moment, we've got a really good system, where we've got a pharmacist... And they come every week to the home, and they just ask us, are there any residents who you would like us to see? Have you got any concerns this

				week? The pharmacist is there also for any medication queries, and we would always ask them." (Care home 1, Deputy manager)
	Inner setting	Access to knowledge and information	Ease of access to digestible information and knowledge about deprescribing and medicines and how to incorporate it into the way staff provide care for residents.	"And awareness of different medications, because as much as we're residential here, so there's no nurses on site, we have the district nursing team. And, obviously, I couldn't tell you what every single medication does, and even to this day, I still have to Google sometimes and I'm like, oh, this is new! If I knew a little bit more about medications and I was aware of more potential side-effects and stuff, then maybe I'd be a little bit more passionate in communicating with the GP, any concerns." (Care home 13, Business manager)<br><br>"We just get told, oh, they've had a blood test, they need to have this medication. And it's like, but what did the blood tests show? And it's like they don't always tell us, and it's just like, really? Give us more information, please!" (Care home 9, Deputy manager)
		Available resources	The level of resources the care home dedicated for making changes to the way things are done and on-going operations including physical space and time.	"But because of Covid, I think a lot of things have been put on the back shelf a little bit, and I don't think the doctors have time. I understand, but they will look at urgent queries, but general medication reviews, I think it's not a vital thing at the moment, because of the last year or so with Covid." (Care home 1, Deputy manager)<br><br>"I think the main thing we need, is time. If I think of the care home I look after, there's 65 residents, so I've got to find... If I want to do it properly, I'd have find at least 65 hours a year to do it regularly, and follow-ups. So it's really quite hard to manage...So time is the biggest problem, and also, and time for care home staff. I mean, they're as pressured as anyone else, for them to actually spend...

For peer review only

				Take an hour of their day out to talk about each one of their residents, again, is quite onerous on them." (Care home 8, GP)
		Networks and communication	The nature and quality of webs of social networks, and the nature and quality of formal and informal communications within the care home	"Because, obviously, it's on the [Medicine Administration Record] sheet, it's crossed off on our [Medicine Administration Record] sheets, stopped by the GP. It's documented in their care plans. It's documented on our [Patient Centred Software]. It's all well-documented, and like you do medication, you have to follow your [Medicine Administration Record] sheets, so it's always there so everyone can see what's stopped and why, and who stopped it and what date." (Care home 15, Head of Care)<br><br>"We write everything down, and we've got behavioural charts, etcetera, and How Was My Day? So even if the head of care was off or whoever was taking… We would have a writing log, a weekly log, daily log of the changes." (Care home 14, Deputy manager)
		Tension for change	The degree to which the current situation as intolerable or needing change	"But we do feel, at times, they are on an awful lot of medication, and unless we ask for a medication review, they don't really get one. So they could be on lots of medications for many, many years without actually a review. So they can actually - it can actually, probably be doing more harm than good, possibly, because some of them take a lot, a lot, a lot of medication." (Care home 10, Care supervisor)<br><br>" So, yeah, I definitely think that they need to be reviewed regularly, because you don't want people taking medication that they no longer need, that they no longer require, if that makes sense?" (Care home 4, Supervisor)
		Resident needs and resources	The extent to which the needs of care home residents, as well as	"But, as I said, we work quite closely and you see changes in those residents. So if there's something that can help them in a different way, and if something is, we feel is not quite working, then that

For peer review only

			barriers and facilitators to meet those needs, are accurately known and prioritized by the care home	would be something that could be stopped, going down the right route." (Care home 14, Deputy manager)<br><br>" So it comes about from us knowing our residents, and being able to make sure we pass that information clearly and accurately and timely onto the surgery, as I mentioned before." (Care home 4, Deputy manager)
	Characteristics of deprescribing (intervention)	Evidence strength and quality	Perceptions of the quality and validity of evidence supporting the belief that deprescribing will have positive outcomes for residents or will do no harm (adverse outcomes).	"I'm aware there is some, but I can't quote anything offhand. We get sent it by our medicines management team quite often, about reviewing. So particularly with antipsychotics...But, yeah, so there is some, and there is our medicines management team at CCG who are really good about supporting us with evidence to things." (Care home 8, GP)
		Cost	Costs of deprescribing and costs associated with implementing deprescribing including investment, supply, and opportunity costs	"The amount of money that must be being spent on medication, to reduce it must be a massive, massive saving."(Care home 4, Deputy manager)<br><br>"I don't think there's any direct cost involved to us, apart from our time. But it would be a nice cost to the resident, because, hopefully, we'd be able to spend more quality time with them, rather than being shove that down your throat, we've got another 40 more to do." (Care home 11, Manager)
		Adaptability	The degree to which deprescribing can be adapted, tailored, refined, or reinvented to meet needs at organisation/provider	"I guess it's you just see - yeah, it's difficult because everyone's being, should be treated as an individual, so therefore they should have their… I'm going to come back to the same point, but regular reviews of their medications, so that their condition at that time,

For peer review only

			(care home) and individual (resident) level	and they're not taking anything unnecessarily, and that's got to be an advantage for them." (Care home 4, Deputy manager)<br><br>"Well, yeah, we're all slightly different. We think we're the same, but we're not and our bodies react differently. But what works - if I come back to this, all these tablets, to my knowledge, work for me." (Care home 14, resident)
		Complexity	Perceived difficulty of the deprescribing	"It's looking for any changes. It's much easier to do this with people who've got capacity, who can tell you. With people with dementia whose communication is limited, you do have to be monitoring more, actually. What are they - are they normal for them? " (Care home 2, Nurse supervisor)<br><br>"Oh, it's very complicated. I mean, we've seen through Covid, a set of mixed messages, really, where the determination is to try and support care homes, through medication optimisation in care homes. And there's been a whole kind of industry at work on that for many years, actually, not just within Covid." (Care home 14, Director)
		Relative advantage	Perception of the outcome deprescribing (i.e. better, worse, no change) versus an alternative solution (i.e. continuing medications).	"They take less tablets, and so then they can feel happier, because they haven't got to take so much medication." (Care home 4, Supervisor)<br><br>"I don't want her to take things that don't do her - don't do anything." (Care home 2, family carer)
		Trialability	The ability to test deprescribing on a small scale in the care home, and to be able to	"Well, if it was clear to the medical people, if they wouldn't be, if it was clear to them, and they could go communicate to me, they

For peer review only

			reverse course (undo implementation) if warranted (i.e. restart medications).	would try and I'd give it a try, as long as I could go back if I wasn't happy." (Care home 1, resident)<br>"Yes, it can, and quite often, if a doctor has asked me to suspend the medication or, I don't know, say for a week, they do usually say, however, this is the things you should look out for. You should look out for A, B and C if this happens, either restart it or contact us again." (Care home 2, Nurse supervisor)

For peer review only

STOPPING

Understanding stakeholders' perspectives on implementing deprescribing in care homes (STOPPING)

TOPIC GUIDE WITH CARE HOME STAFF AND HEALTHCARE PROFESSONALS

Introduction

We are here today to talk about your experiences with and views of deprescribing (stopping a medication or reducing its dosage) in care homes. The purpose is to get your perceptions of how reducing or stopping some medicines would work in a care home setting so we can identify the key barriers, facilitators and contextual factors influencing it. I am not here to share information, or to give you my opinions. Your perceptions are what matter. There are no right or wrong or desirable or undesirable answers. You can disagree with each other, and you can change your mind. I would like you to feel comfortable saying what you really think and how you really feel.

As you know everything is confidential.

Topics

Knowledge & beliefs about the intervention

What do you think about reducing or stopping some medicines for people living in care homes?

Probes. Why do you think that? Do you have any concerns? Are there any benefits?

Do you think reducing or stopping some medicines will be beneficial in your care home?

Probes. Why or Why not? Do you have any concerns?

Relative advantage

Do you think are the advantages of reducing or stopping some medicines for people living in care homes compared to continuing their medicines?

Probes. Why? Is that your experience? Anything else?

Do you think are the disadvantages of reducing or stopping some medicines for people living in care homes compared to continuing their medicines?

Probes. Why? Is that your experience? Anything else?

Tension for change

Is there a strong need for reducing or stopping some medicines?

Probes. Why or why not? Do others see a need for it?

How essential is this reducing or stopping some medicines to meet the needs of the individuals served by your care home?

How do you feel about current programs/practices/process that are available?

Probes. What do they do well? To what extent do current programs fail to meet existing needs?

Complexity

How complicated is it to reduce or stop some medicines for care home residents?

Probes. Please consider the following aspects: duration, scope, intricacy and number of steps involved

For peer review only

STOPPING

Trialability

What do you think about trying to stop or reduce a medicine (or medicines) for a short period?

Probes. What would that be like? Would it be helpful? Harmful? Why or why not?

Evidence strength & quality

What kind of information or evidence are you aware of that shows whether or not the reducing or stopping some medicines will work in your care home?

Probes. What evidence have you heard about? Practice guidelines? Published literature? Co-workers? Other care homes? How does this knowledge affect your view?

What kind of supporting evidence or proof is needed about the effectiveness of reducing or stopping some medicines to get staff on board?

Probes. Co-workers? Administrative leaders?

Planning

What needs to be done beforehand so that reducing or stopping medicines can happen in care homes?

Probes. Why? Is that your experience? What steps should be included? Anything else? What is your role in the planning?

Engaging

What do you think the role of the care home and its staff should be in reducing and stopping some medicines?

Probes. How would that like look? To what extent?

What do you think the role of the GPs should be?

Probes. How would that like look? To what extent?

What do you think the role of the pharmacist should be?

Probes. How would that like look? To what extent?

Is there anyone else who should be involved?

Probes. How would that like look? To what extent?

Reflecting & Evaluating

What do you think should be the outcome of reducing or stopping some medicines for people living in care homes?

Probes. Why? Is that your experience? Anything else?

What should happen after reducing or stopping some medicines?

Probes. What information should be collected? How often? Feedback given?

Communication & Network

How do you know when there have been changes to the resident's medicines?

Probes. What gets in the way of knowing? Can this be improved? If so how?

For peer review only

STOPPING

Can you describe your working relationships with your colleagues?

Probes. With colleagues in your care home? With colleagues in other care homes? Can you tell me a story about a time you needed to work with others to solve a problem? Or to implement reducing to stopping a resident's medicines?

Do you understand why there have been changes to a resident's medicines?

Probes. Why? What would aid your understanding? What stops you from understanding? How could this be communicated better?

Cosmopolitanism

What kind of information sharing do you have with other professionals and organisations outside your care home, either related to the medicines, or more generally?

Probes. What professional or care home networking do you engage in? Listservs? Local or national conferences? Trainings?

Executing & Personal Attributes

Please tell me about any experiences that you may have with stopping or reducing some of the residents' medicines.

Probes. How did it go? What happened? When been done well? When has it not gone so well? What would help work better in the future? Why was that the case? Was anything done by the care home staff that helped this experience? Or impeded it? What problems or issues did you experience, if any?

Available resources

Do you expect to have sufficient resources to implement and administer reducing or stopping some medicines?

Probes. [If Yes] What resources are you counting on? Are there any other resources that you received, or would have liked to receive? What resources will be easy to procure?
[If no] What resources will not be available?

Cost

What are the costs associated with reducing or stopping a resident's medicines?

Probes. What costs will be incurred? What cost were considered when deciding to implement?

Patient needs & resources

How do the residents' needs and preferences get included in decisions to change their medicines?

Probes. Why were they included or not included? How 'in touch' were the people involved? Were they prioritised over other things? Has anything been altered or changed to meet the resident's needs? What would you like your input to be?

How would you feel about stopping or reducing some of the resident's medicines?

Probes. Is there anything that would prevent you? Why?

Self-efficacy

Do you think you can question the amount of medicines that residents are taking?

For peer review only

STOPPING

Probes. How confident are you to do this? Would you do it? Have you? Why or Why not?

Closure

Is there any other information regarding your experiences or opinions that you think would be useful for me to know?

Thank you very much for meeting with me. Your time is very much appreciated and your comments have been very helpful.

For peer review only

STOPPING

Understanding stakeholders' perspectives on implementing deprescribing in care homes (STOPPING)

INTERVIEW TOPIC GUIDE WITH CARE HOME RESIDENT

Introduction

Good morning/afternoon. My name is Krystal Warmoth. I am going to ask you some questions about reducing or stopping some prescription medicines. For example, someone may need to stop taking a medicine that may no longer be providing benefit or has side effects (like falling) as they get older, or reducing the dose of a medication so that the person doesn't have to take as many tablets each day. We still don't really know how to make it work in real-life settings, like care homes. In this project, we aim to study how reducing or stopping some medicines can be best done in care homes, considering different views and settings.

We are not wanting to change your medicines, we are just wanting to chat with you to understand what happens at the moment, in terms of how your medicines may or may not be stopped or reduced. And discuss your thoughts on this happening, and how we could one day, make sure it's done well.

Again, I want to remind you that your responses will be kept confidential and you do not have to answer any question if you do not wish to. You can also stop the interview at any time.

Before we start, I want to check that you are happy to be interviewed and for the interview to be recorded.

Interview

1. *To start, I want to know what your opinions are about reducing or stopping some medicines. What do you think about the idea of reducing or stopping some medicines?* ***Knowledge & beliefs***

   Probes. Why do you think that? Do you have any concerns? Are there any benefits?

2. *Do you think there are the* advantages of reducing or stopping some medicines*?* ***Knowledge & beliefs & Relative advantage***

   Probe. Why or Why not? Do you think are the disadvantages? Why or Why not?

3. *What do you think about trying to stop or reduce a medicine (or medicines) for a short period of time?* ***Trialability***

   Probes. What would that be like? Who would lead it? Would it be helpful? Harmful? Why or why not?

4. *What things will need to happen when planning to reduce or stop some medicines?* ***Planning***

   Probes. Why? Is that your experience? What steps should be included? Anything else? What is your role in the planning?

[CHECK IF THEY ARE HAPPY TO CONTINUE OR WOULD LIKE TO TAKE A BREAK]

5. *Do you think different approaches to reducing or stopping some medicines would be needed for different people?* ***Adaptability***

   Probes. Why or why not? What kinds of changes do you think would need to be made? Do you think these changes are possible? Why or Why not?

For peer review only

STOPPING

6. *What do you think the role of the care home and its staff should be in reducing and stopping some medicines?* ***Engaging***

   Probes. How would that like look? To what extent?

7. *What do you think the role of the GPs should be in reducing and stopping some medicines?* ***Engaging***

   Probes. How would that like look? To what extent?

8. *What do you think the role of the pharmacist should be in reducing and stopping some medicines?* ***Engaging***

   Probes. How would that like look? To what extent?

9. *Is there anyone else that needs to be involved in how to reduce or stop some medicines?* ***Engaging***

   Probes. Why? How would that like look? To what extent? What about family and friends?

[CHECK IF THEY ARE HAPPY TO CONTINUE OR WOULD LIKE TO TAKE A BREAK]

10. *What do you think should be the impact or end result of reducing or stopping some medicines for people living in care homes?* ***Reflecting & Evaluating***

    Probes. Why? Is that your experience? Anything else? What about quality of life? Side effects?

11. *Should anything else happen after someone's medicines have been reduced or stopped?* ***Reflecting & Evaluating***

    Probes. What information should be collected? How often? Feedback given?

12. *Did you know when there have been changes to your medicines?* ***Communication & Network***

    Probes. How did you know? What gets in the way of knowing?

13. *Did you understand why there were changes to your medicines?* ***Knowledge & beliefs***

    Probes. Why? What would aid your understanding? What stops you from understanding? How could this be communicated better?

[CHECK IF THEY ARE HAPPY TO CONTINUE OR WOULD LIKE TO TAKE A BREAK]

14. *Now, I would like to know about your own experiences with reducing or stopping some medicines. Please tell me about any experiences that you may have with stopping or reducing some of your medicines while you [they] have been living in the care home.* ***Executing & Personal Attributes***

    Probes. How did it go? What happened? When has it been done well? When has it not gone so well? What would help it work better in the future? Why was that the case? Was anything done by the care home staff that helped this experience? Or impeded it? What problems or issues did you experience, if any?

[IF CANNOT REMEMBER AN EXPERIENCE -> SKIP TO QUESTION 16]

[CHECK IF THEY ARE HAPPY TO CONTINUE OR WOULD LIKE TO TAKE A BREAK]

15. *Thinking back to your experience of medicines being stopped or reduced. How did your needs and preferences get included in decisions to change your medicines?* ***Patient needs & resources***

For peer review only

STOPPING

Probes. Why were they included or not included? How 'in touch' were the people involved? Were they prioritised over other things? Has anything been altered or changed to meet your needs? What would you like your input to be?

16. *Do you think you can question the amount of medicines that you are taking?* ***Self-efficacy***

    Probes. How confident are you to do this? Would you do it? How would you talk to about this? Have you? Why or Why not?

17. How would you feel about stopping or reducing some of your medicines? ***Patient needs & resources***

    Probes. Would you do it? Is there anything that would prevent you doing it? Why?

Closure

Is there any other information regarding your opinions or experiences that you think would be useful for me to know?

Again, I want to let you know that your medicines are not being changed based on what we have talked about. If you wish to change your medications as a result of this discussion, we will let the care home staff know so you can discuss this with your general practitioner and any other relevant people. Please do not change your medicines until you have talked with the appropriate healthcare professional.

Thank you very much for meeting with me. Your time is very much appreciated and your comments have been very helpful.

To finish, I am going to ask a few questions to get some basic information about you.

For peer review only

STOPPING

Understanding stakeholders' perspectives on implementing deprescribing in care homes (STOPPING)

INTERVIEW TOPIC GUIDE WITH FAMILY OR FRIEND

Introduction

Good morning/afternoon. My name is Krystal Warmoth. I am going to ask you some questions about reducing or stopping some prescription medicines. For example, someone may need to stop taking a medicine that may no longer be providing benefit or has side effects (like falling) as they get older, or reducing the dose of a medication so that the person doesn't have to take as many tablets each day. We still don't really know how to make it work in real-life settings, like care homes. In this project, we aim to study how reducing or stopping some medicines can be best done in care homes, considering different views and settings.

We are not wanting to change [INSERT RESIDENT'S NAME]'s medicines, we are just wanting to chat with you to understand what happens at the moment, in terms of how the [INSERT RESIDENT'S NAME]'s medicines may or may not be stopped or reduced. And discuss your thoughts on this happening, and how we could one day, make sure it's done well.

Again, I want to remind you that your responses will be kept confidential and you do not have to answer any question if you do not wish to. You can also stop the interview at any time.

Before we start, I want to check that you are happy to be interviewed and for the interview to be recorded.

Interview

18. *To start, I want to know what your opinions are about reducing or stopping some medicines. What do you think about the idea of reducing or stopping some medicines? **Knowledge & beliefs***

    Probes. Why do you think that? Do you have any concerns? Are there any benefits?

19. *Do you think there are the* advantages of reducing or stopping some medicines*? **Knowledge & beliefs & Relative advantage***

    Probe. Why or Why not? Do you think are the disadvantages? Why or Why not?

20. *What do you think about trying to stop or reduce a medicine (or medicines) for a short period of time? **Trialability***

    Probes. What would that be like? Who would lead it? Would it be helpful? Harmful? Why or why not?

21. *What things will need to happen when planning to reduce or stop some medicines? **Planning***

    Probes. Why? Is that your experience? What steps should be included? Anything else? What is your role in the planning?

[CHECK IF THEY ARE HAPPY TO CONTINUE OR WOULD LIKE TO TAKE A BREAK]

22. *Do you think different approaches to reducing or stopping some medicines would be needed for different people living in a care home? **Adaptability***

    Probes. Why or why not? What kinds of changes do you think would need to be made? Do you think these changes are possible? Why or Why not?

For peer review only

STOPPING

23. *What do you think the role of the care home and its staff should be in reducing and stopping some medicines? **Engaging***

Probes. How would that like look? To what extent?

24. *What do you think the role of the GPs should be in reducing and stopping some medicines? **Engaging***

Probes. How would that like look? To what extent?

25. *What do you think the role of the pharmacist should be in reducing and stopping some medicines? **Engaging***

Probes. How would that like look? To what extent?

26. *Is there anyone else that needs to be involved in how to reduce or stop some medicines? **Engaging***

Probes. Why? How would that like look? To what extent? What about family and friends?

[CHECK IF THEY ARE HAPPY TO CONTINUE OR WOULD LIKE TO TAKE A BREAK]

27. *What do you think should be the impact or end result of reducing or stopping some medicines for people living in care homes? **Reflecting & Evaluating***

Probes. Why? Is that your experience? Anything else? What about quality of life? Side effects?

28. *Should anything else happen after someone's medicines have been reduced or stopped? **Reflecting & Evaluating***

Probes. What information should be collected? How often? Feedback given?

29. *Did you know when there have been changes to [INSERT RESIDENT'S NAME]'s medicines? **Communication & Network***

Probes. How did you know? What gets in the way of knowing?

30. *Did you understand why there were changes to [INSERT RESIDENT'S NAME]'s medicines? **Knowledge & beliefs***

Probes. Why? What would aid your understanding? What stops you from understanding? How could this be communicated better?

[CHECK IF THEY ARE HAPPY TO CONTINUE OR WOULD LIKE TO TAKE A BREAK]

31. *Now, I would like to know about your own experiences with reducing or stopping some medicines. Please tell me about any experiences that you may have with stopping or reducing some of [INSERT RESIDENT'S NAME]'s medicines while they have been living in the care home. **Executing & Personal Attributes***

Probes. How did it go? What happened? When has it been done well? When has it not gone so well? What would help it work better in the future? Why was that the case? Was anything done by the care home staff that helped this experience? Or impeded it? What problems or issues did you experience, if any?

[IF CANNOT REMEMBER AN EXPERIENCE -> SKIP TO QUESTION 16]

[CHECK IF THEY ARE HAPPY TO CONTINUE OR WOULD LIKE TO TAKE A BREAK]

For peer review only

STOPPING

32. *Thinking back to your experience of medicines being stopped or reduced. How did your needs and preferences get included in decisions to change [INSERT RESIDENT'S NAME]'s medicines?* ***Patient needs & resources***

    Probes. Why were they included or not included? How 'in touch' were the people involved? Were they prioritised over other things? Has anything been altered or changed to meet your [resident's] needs? What would you like your input to be?

33. *Do you think you can question the amount of medicines that [INSERT RESIDENT'S NAME] are taking?* ***Self-efficacy***

    Probes. How confident are you to do this? Would you do it? How would you talk to about this? Have you? Why or Why not?

34. How would you feel about stopping or reducing some of *[INSERT RESIDENT'S NAME]'s* medicines? ***Patient needs & resources***

    Probes. Would you do it? Is there anything that would prevent you doing it? Why?

Closure

Is there any other information regarding your opinions or experiences that you think would be useful for me to know?

Again, I want to let you know that your [resident's] medicines are not being changed based on what we have talked about. If you wish to change your [resident's] medications as a result of this discussion, we will let the care home staff know so you can discuss this with your general practitioner and any other relevant people. Please do not change your [resident's] medicines until you have talked with the appropriate healthcare professional.

Thank you very much for meeting with me. Your time is very much appreciated and your comments have been very helpful.

To finish, I am going to ask a few questions to get some basic information about you.

For peer review only

COREQ (COnsolidated criteria for REporting Qualitative research) Checklist

A checklist of items that should be included in reports of qualitative research. You must report the page number in your manuscript where you consider each of the items listed in this checklist. If you have not included this information, either revise your manuscript accordingly before submitting or note N/A.

Topic	Item No.	Guide Questions/Description	Reported on Page No.
**Domain 1: Research team and reflexivity**			
Personal characteristics			
Interviewer/facilitator	1	Which author/s conducted the interview or focus group?	
Credentials	2	What were the researcher's credentials? E.g. PhD, MD	
Occupation	3	What was their occupation at the time of the study?	
Gender	4	Was the researcher male or female?	
Experience and training	5	What experience or training did the researcher have?	
Relationship with participants			
Relationship established	6	Was a relationship established prior to study commencement?	
Participant knowledge of the interviewer	7	What did the participants know about the researcher? e.g. personal goals, reasons for doing the research	
Interviewer characteristics	8	What characteristics were reported about the inter viewer/facilitator? e.g. Bias, assumptions, reasons and interests in the research topic	
**Domain 2: Study design**			
Theoretical framework			
Methodological orientation and Theory	9	What methodological orientation was stated to underpin the study? e.g. grounded theory, discourse analysis, ethnography, phenomenology, content analysis	
Participant selection			
Sampling	10	How were participants selected? e.g. purposive, convenience, consecutive, snowball	
Method of approach	11	How were participants approached? e.g. face-to-face, telephone, mail, email	
Sample size	12	How many participants were in the study?	
Non-participation	13	How many people refused to participate or dropped out? Reasons?	
Setting			
Setting of data collection	14	Where was the data collected? e.g. home, clinic, workplace	
Presence of non-participants	15	Was anyone else present besides the participants and researchers?	
Description of sample	16	What are the important characteristics of the sample? e.g. demographic data, date	
Data collection			
Interview guide	17	Were questions, prompts, guides provided by the authors? Was it pilot tested?	
Repeat interviews	18	Were repeat inter views carried out? If yes, how many?	
Audio/visual recording	19	Did the research use audio or visual recording to collect the data?	
Field notes	20	Were field notes made during and/or after the inter view or focus group?	
Duration	21	What was the duration of the inter views or focus group?	
Data saturation	22	Was data saturation discussed?	
Transcripts returned	23	Were transcripts returned to participants for comment and/or	

For peer review only

Topic	Item No.	Guide Questions/Description	Reported on Page No.
		correction?	
**Domain 3: analysis and findings**			
Data analysis			
Number of data coders	24	How many data coders coded the data?	
Description of the coding tree	25	Did authors provide a description of the coding tree?	
Derivation of themes	26	Were themes identified in advance or derived from the data?	
Software	27	What software, if applicable, was used to manage the data?	
Participant checking	28	Did participants provide feedback on the findings?	
Reporting			
Quotations presented	29	Were participant quotations presented to illustrate the themes/findings? Was each quotation identified? e.g. participant number	
Data and findings consistent	30	Was there consistency between the data presented and the findings?	
Clarity of major themes	31	Were major themes clearly presented in the findings?	
Clarity of minor themes	32	Is there a description of diverse cases or discussion of minor themes?	

Developed from: Tong A, Sainsbury P, Craig J. Consolidated criteria for reporting qualitative research (COREQ): a 32-item checklist for interviews and focus groups. *International Journal for Quality in Health Care*. 2007. Volume 19, Number 6: pp. 349 – 357

Once you have completed this checklist, please save a copy and upload it as part of your submission. DO NOT include this checklist as part of the main manuscript document. It must be uploaded as a separate file.

For peer review only

BMJ Open

DETERMINANTS OF IMPLEMENTING DEPRESCRIBING FOR OLDER ADULTS IN ENGLISH CARE HOMES: A QUALITATIVE INTERVIEW STUDY

Journal:	*BMJ Open*
Manuscript ID	bmjopen-2023-081305.R1
Article Type:	Original research
Date Submitted by the Author:	01-Nov-2023
Complete List of Authors:	Warmoth, Krystal ; University of Hertfordshire, Centre for Research in Public Health and Community Care; National Institute for Health Research Applied Research Collaboration East of England<br>Rees, Jessica; King's College London, Institute of Gerontology<br>Day, Jo; University of Exeter, Health and Community Sciences; National Institute for Health Research Applied Research Collaboration South West Peninsula<br>Cockcroft, Emma; University of Exeter Medical School, Clinical Trials Unit; National Institute for Health Research Applied Research Collaboration South West Peninsula<br>Aylward, Alex; Patient and Public Involvement Group<br>Pollock, Lucy; Somerset NHS Foundation Trust<br>Coxon, George; Classic Care (Devon) Homes Ltd<br>Craig, Trudy; Somerset Care Ltd<br>Walton, Bridget; National Institute for Health Research Applied Research Collaboration South West Peninsula<br>Stein, Ken; University of Exeter Medical School, Health and Community Sciences; National Institute for Health Research Applied Research Collaboration South West Peninsula
**Primary Subject Heading**:	Geriatric medicine
Secondary Subject Heading:	Health services research
Keywords:	Implementation Science, QUALITATIVE RESEARCH, Health Services for the Aged, Patient-Centered Care

SCHOLARONE™
Manuscripts

I, the Submitting Author has the right to grant and does grant on behalf of all authors of the Work (as defined in the below author licence), an exclusive licence and/or a non-exclusive licence for contributions from authors who are: i) UK Crown employees; ii) where BMJ has agreed a CC-BY licence shall apply, and/or iii) in accordance with the terms applicable for US Federal Government officers or employees acting as part of their official duties; on a worldwide, perpetual, irrevocable, royalty-free basis to BMJ Publishing Group Ltd ("BMJ") its licensees and where the relevant Journal is co-owned by BMJ to the co-owners of the Journal, to publish the Work in this journal and any other BMJ products and to exploit all rights, as set out in our licence.

The Submitting Author accepts and understands that any supply made under these terms is made by BMJ to the Submitting Author unless you are acting as an employee on behalf of your employer or a postgraduate student of an affiliated institution which is paying any applicable article publishing charge ("APC") for Open Access articles. Where the Submitting Author wishes to make the Work available on an Open Access basis (and intends to pay the relevant APC), the terms of reuse of such Open Access shall be governed by a Creative Commons licence – details of these licences and which Creative Commons licence will apply to this Work are set out in our licence referred to above.

Other than as permitted in any relevant BMJ Author's Self Archiving Policies, I confirm this Work has not been accepted for publication elsewhere, is not being considered for publication elsewhere and does not duplicate material already published. I confirm all authors consent to publication of this Work and authorise the granting of this licence.

For peer review only

DETERMINANTS OF IMPLEMENTING DEPRESCRIBING FOR OLDER ADULTS IN ENGLISH CARE HOMES: A QUALITATIVE INTERVIEW STUDY

Krystal Warmoth1,2*, Jessica Rees1,2, Jo Day3,4, Emma Cockcroft3,4, Alex Aylward5, Lucy Pollock6, George Coxon7, Trudy Craig8, Bridget Walton4, & Ken Stein3,4

1 Centre for Research in Public Health and Community Care, University of Hertfordshire, Hatfield, UK

2 National Institute for Health Research Applied Research Collaboration East of England, Cambridge, UK

3 Health and Community Sciences, University of Exeter, Exeter, UK

4 National Institute for Health Research Applied Research Collaboration South West Peninsula, Exeter, UK

5 Peninsula Public Involvement Group, National Institute for Health Research Applied Research Collaboration South West Peninsula, Exeter, UK

6 Musgrove Park Hospital, Somerset NHS Foundation Trust, Taunton, UK

7 Classic Care (Devon) Homes Ltd., Teignmouth, UK

8 Somerset Care Ltd., Taunton, UK

* Corresponding author: Krystal Warmoth, PhD. University of Hertfordshire, Centre for Research in Public Health and Community Care, Health Research Building, College Lane Campus, Hatfield, UK, AL10 9AB; Email: k.warmoth@herts.ac.uk

For peer review only

Abstract

Objectives: To explore the factors that may help or hinder deprescribing practice for older people within care homes.

Design: Qualitative semi-structured interviews using framework analysis informed by the Consolidated Framework for Implementation Research.

Setting: Participants were recruited from two care home provider organisations (a smaller independently-owned organisation and a large organisation) in England.

Participants: A sample of 23 care home staff, 8 residents, 4 family members, and 1 general practitioner (GP) were associated with 15 care homes.

Results: Participants discussed their experiences and perceptions of implementing deprescribing within care homes. Major themes of (1) deprescribing as a complex process and (2) internal and external contextual factors influencing deprescribing practice (such as beliefs, abilities, and relationships) were interrelated and spanned several Consolidated Framework for Implementation Research constructs and domains. The quality of local relationships with and support from healthcare professionals were considered more crucial factors than the type of care home management structure.

Conclusions: Several influencing social and contextual factors need to be considered for implementing deprescribing for older adults in care homes. Additional training, tools, support, and opportunities need to be made available to care home staff so they can feel confident and able to question or raise concerns about medicines with prescribers. Further work is warranted to design and adopt a deprescribing approach which addresses these determinants to ensure successful implementation.

Keywords: Polypharmacy, Medicine optimisation, Implementation science, Qualitative Study, Older adults, Homes for the Aged

For peer review only

Strengths and limitations of this study

- Data collection and analysis were informed by a comprehensive, well-recognised implementation science framework.
- This research adopted a strong patient and public involvement and partnership approach.
- The number of respondents who participated during the pandemic and the high demand for healthcare services should be acknowledged.
- Majority of participants were care home staff so the findings reflect mostly their perspectives and experiences.
- Methods were modified from the original protocol due to the COVID-19 pandemic.

Word count 4190

For peer review only

Introduction

Older people living in UK care homes often experience polypharmacy, which is defined as receiving five or more concurrent medicines a day (1). In England, the 2021 National Overprescribing Review found older people to be at greater risk from polypharmacy; over half of the people over the age of 80 take eight or more medicines a day (2), many of whom live in care homes. Polypharmacy is widespread, with over 60% of residents taking five or more medicines (3), and increasing with system-wide overprescribing (2, 4). Some older adults are prescribed multiple medicines that are unlikely to improve clinical outcomes, are clinically unnecessary, or may lead to harm (5-8). One-half of care home residents are exposed to potentially inappropriate medicines (9). Care home staff, residents and family members stress the high prevalence, fears about the health and safety consequences, and burden of polypharmacy in care homes (10).

Reducing or stopping prescription medicines which may no longer be providing benefit or where the harms outweigh the benefits, known as deprescribing (11), can mitigate these harms and be safe (12-15). National Institute for Health and Care Excellence (NICE) recommends deprescribing as part of the comprehensive medication review of a person with multiple long-term conditions (16-18). While appropriate deprescribing is usually commended and may be cautiously undertaken to good effect (15), there is a lack of information about how to implement it safely and appropriately (19). Recommendations overlook the specific contextual factors and various stakeholder views of care homes (20). Deprescribing in this setting can be challenging due to the different concerns of residents, staff, clinicians, and family members and differences in care home structures.

Previous deprescribing research explored generic views towards barriers and facilitators to deprescribing (e.g., 21, 22-27) and only focused on the perspectives of a single stakeholder group, such as patients (e.g., 25, 27) or general practitioners (e.g., 22, 23, 24),

For peer review only

and rarely included crucial care home staff views (28). Different stakeholder groups from long-term care facilities prioritise different factors (29). There is a need for a better understanding of the factors influencing how deprescribing is implemented considering different stakeholders.

A recent summary of the perceptions of deprescribing in long-term care facilities from nine different countries found social influences and environmental factors were perceived as the key barriers and enablers (30). Considering this, the present study investigated the process of deprescribing and these contextual factors in English care homes, including the roles and relationships of different stakeholders, which influence it. Furthermore, implementation activities of deprescribing in care homes are typically not well-described and poorly understood (19, 31). A better understanding of how contextual factors facilitate or hinder deprescribing, informed by an implementation framework, is needed to support the translation of deprescribing recommendations into practice (32, 33).

The present paper presents the findings of the first work package of the STOPPING project (for the original protocol, see 34). The overall aim was to investigate the factors which influence deprescribing for older adults in care homes. The specific objectives of the research were to:

1. Identify the factors which influence deprescribing for older adults in care homes;
2. Explore factors influencing deprescribing across a variety of stakeholders and care home organisations; and
3. Use the Consolidated Framework for Implementation Research (CFIR) to help understand the possible determinants of implementing deprescribing for older adults in a care home setting.

For peer review only

Methods

Design

We used qualitative methods and adopted a pragmatic approach. Originally, focus groups with care home staff and healthcare professionals and in-person observations of care homes were planned but were no longer feasible due to the COVID-19 pandemic. The original methods were changed to allow for remote data collection. Individual or dyadic interviews were conducted with all participants via Microsoft® Teams, Zoom, or by telephone.

We gathered and compared perspectives from various stakeholders from two different types of care home provider organisations and used an implementation science framework to inform the data collection and analysis. The CFIR was used as an overarching framework to ensure that implementation was central to data collection and analysis (35). CFIR (2009 version) is a well-established, theoretically based implementation science framework, comprised of 39 constructs divided into five domains (35). CFIR focuses on identifying and understanding constructs that can shape the implementation and the routinisation of health services (36), making it appropriate for care homes.

Setting

The two care home providers were selected for this research as they represent contrasting models of care home provision. That is, one is a smaller independently-owned organisation consisting of two residential care home sites and a large organisation with more than 25 care homes in several locations across England. They were also selected as they mainly care for older adults (over 65 years old). Care home sites included in this study were selected in partnership between the research team and senior care home staff.

For peer review only

Participants

Recruitment and data collection occurred from December 2021 to May 2022. Table 1 for participant details and Table 2 for the care home details. Care home and other healthcare staff were eligible for the study if they were currently working directly with older adults with polypharmacy and/or in the care home setting and could converse in English without an interpreter or professional assistance. Participants were purposefully recruited from distinct types of care home providers using established networks of care home organisations. Care home staff were selected and approached by the regional managers or directors of the care home organisations, who were known to the researchers.

For peer review only

Table 1. Participant characteristics (n= 36)

	n (%)	M (SD)
**Care home staff and health care professionals (n=24)**		
Age		44.65 (12.04)
Gender		
Female	21 (87.50)	
Male	3 (12.50)	
Ethnic group		
White	23 (95.80)	
Asian	1 (4.20)	
Education		
General Certificate of Education	3 (12.50)	
Undergraduate degree	5 (20.8 0)	
National vocational qualification	14 (58.30)	
Postgraduate degree	2 (8.30)	
Employment duration (in months)		47.83 (59.27)
Key responsibilities and duties		
Assistance with daily tasks in care home	14 (58.33)	
Administering medicines	17 (70.83)	
Providing company and assistance in leisure activities	5 (20.83)	
Developing care plans	20 (83.33)	
Planning/overseeing work of other staff members	21 (87.50)	
Prescribing and medicine review	16 (66.67)	
Other	14 (58.33)	
Hours worked per week		
Less than 32 hours	3 (12.50)	
More than 32 hours	21 (87.50)	
**Family carers (n=4)**		

Age		64.75 (1.26)
Gender		
Female	3 (75.00)	
Male	1 (25.00)	
Ethnic group - White	4 (100.00)	
Relationship to resident		
Son	1 (25.00)	
Daughter	2 (50.00)	
Sibling	1 (25.00)	
**Residents (n=8)**		
Age		88.75 (5.06)
Gender		
Female	6 (75.00)	
Male	2 (25.00)	
Ethnic group - White	8 (100.00)	
Perceived health status		3.50 (0.93)
1. Excellent	1 (12.50)	
2. Very good	0	
3. Good	3 (37.50)	
4. Fair	3 (37.50)	
5. Poor	1 (12.50)	
Number medicines daily		7.71 (3.99)
Number of conditions		6.40 (2.30)

Note. One participant could not remember the number of medicines they took daily so they were not included in the analysis for that item.

Table 2. Care home characteristics

	Number of participants	Number of beds	Occupancy (%)	Total number staff	Dementia speciality	Nursing care
**Large care home provider**						
Care home 1	2 residents, 1 staff	60	88	85	Yes	No
Care home 2	2 family	90	89	140	Yes	Yes
Care home 3	2 staff, 1 family	55	100	75	Yes	Yes
Care home 4	2 staff	34	75	36	No	No
Care home 5	1 staff, 1 resident, 1 family	54	81	57	No	No
Care home 6	1 staff, 1 resident	50	76	32	Yes	No
Care home 7	1 staff	68	83	74	Yes	No
Care home 8	2 staff	46	85	62	Yes	No
Care home 9	1 staff	44	68	36	Yes	No
Care home 10	3 staff, 2 residents	37	91	54	Yes	No
Care home 11	1 staff,	40	95	49	No	No

For peer review only

	1 resident					
Care home 12	2 staff	38	60	42	No	No
Care home 13	1 staff	39	79	50	Yes	No
**Small independent owned provider**						
Care home 14	3 staff, 1 resident	17	100	25	Yes	No
Care home 15	2 staff	19	100	26	Yes	No

Care home staff approached residents and their family members about taking part in the study. The eligibility criteria for care home residents taking part in the research were being a resident of the participating care home, aged 65 and over, taking multiple medications or having experience of polypharmacy, ability to converse in English without an interpreter or professional assistance, and having an absence of serious cognitive impairment, as identified by the care home staff or healthcare profession, and have capacity to consent to participation. Care home residents' family members or friends were eligible if their resident was taking multiple medications at the care home participating in the study and ability to converse in English without an interpreter or professional assistance. Residents with severe cognitive impairment which inhibits consent were excluded, but their family members and carers were included, as this group are particularly at risk of overall drug burden and often benefit from deprescribing interventions.

Data collection

Demographic data were collected from all participants following the interview with a brief survey. Semi-structured interviews were conducted with residents, their family members, care home staff, and healthcare professionals about their experiences and beliefs to identify the factors influencing deprescribing and the current deprescribing practice in different care home settings. Interview topic guides were informed by CFIR constructs identified in previous literature (e.g., 21, 25-27). See Supplementary file 1 for the interview guides. Interviews were conducted by a qualified female researcher with more than 10 years

For peer review only - http://bmjopen.bmj.com/site/about/guidelines.xhtml

experience (KW) and audio-recorded and professionally transcribed verbatim for analysis. Reflective notes were taken to supplement the transcripts. NVivo 12 was used to manage the data.

Qualitative data collection was conducted until data saturation occurred, meaning that no new ideas are being generated. The sample size was determined using previous literature on qualitative methods and using purposeful sampling. We estimated the minimum sample size of 24 was needed, based on work that found data saturation and variability to be present as early as six interviews (37).

Data analysis

A Framework Analysis approach (38) was employed for data analysis. A CFIR-informed codebook was adapted to address the research question and to consider distinct levels of analysis (individual, organisational and community); see Supplementary file 2. Transcripts were deductively coded within the domains and constructs of CFIR by a single researcher (JR). Then, researchers (KW and JR) developed the major themes across the domains inductively. Discussions with patient and public involvement (PPI) representatives helped to refine the major themes and the implications of the research.

Patient and public involvement

This research adopted a strong patient and public involvement and partnership approach with residents, staff, and family carers. Before data collection, a PPI workshop was held with care home residents and staff at one care home. This workshop informed the production of study materials and ensured they were appropriate for and understandable by care home residents and staff. A pilot interview with an independent member of a local PPI group was conducted, and as a result, opportunities for breaks were included and minor changes to the wording of the topic guides were made.

For peer review only

Researchers (JR & KW) and a PPI representative (AA) met regularly to discuss the analysis plan, review and refine the codebook, develop the themes, and interpret the findings.

A final PPI workshop was held to discuss the findings and refine the dissemination strategy with a group of family carers and care home workers. Two researchers and the PPI representative presented the findings and facilitated group discussions about the interpretation of the findings and dissemination. Attendees stated how they shared similar experiences with participants and the varied procedures in care homes.

Results

In total, 36 interviews were conducted with 23 care home staff, 8 residents, 4 family members, and 1 general practitioner (GP) who were associated with 15 care homes. Interviews lasted on average approximately 41 minutes (ranging from 16 to 94 minutes). Two major themes were developed: 1) *Deprescribing as a complex process* and *2) Internal and external contextual factors* influencing deprescribing in care homes. These themes were interrelated and spanned several CFIR constructs and domains. See Table 3 for the major themes, CFIR determinants, and supporting quotes.

For peer review only

Table 3. Summary of factors influencing the implementation of deprescribing in care homes from interviews with residents, family carers, care home staff and healthcare professionals

Major themes	CFIR domains	CFIR constructs	Definitions	Supporting quotes
Deprescribing as a process	Process	Planning	Degree to which tasks or behaviours for implementing deprescribing are developed in advance	"I think there'd be a bit of a process involved. I think probably the family members and staff, the people that know the individuals well, or better than anybody else, they'd have to have an input for their opinion. And then the person that prescribes the medicines, would have to review it and decide what they think for the best." (Care home 14, care assistant)
	Engaging	External change agents	Individuals who are affiliated with organisations/services outside the care home who formally influence or facilitate deprescribing.	"The pharmacist poking his nose in may be good, or it may not be, I don't know." (Care home 14, resident)
		Key stakeholders	Care home staff roles that influence deprescribing	"So I think they're essential for coordinating everyone, really, and actually they're better at doing that than the GP will ever be." (Care home 8, GP)
		Innovation participants - Resident	Resident role in deprescribing (incl. how needs and preferences and included in decisions, and engagement and involvement examples/challenges)	"The resident themselves; they should have an input if they have capacity to do it, if they're able to retain the information about their medication." (Care home 12, Deputy manager)
		Innovation participants -Family carer	Family carer role in deprescribing (incl. how needs and preferences and included in decisions, and engagement and involvement examples/challenges)	"And if they lack capacity, even if they don't lack capacity, then their family, close family, next to kin, power of attorney, we would ask and consult them as well, for anything that changes with any of their care." (Care home 6, Deputy manager and medication lead)
	Reflecting and evaluating		Feedback about the progress and quality of deprescribing and monitoring following deprescribing	"Well, I suppose a final review, at some point, so that's predetermined and there should be a point where it's agreed that there should be a review of how it was effective, but whether it's worked, I suppose." (Care home 2, family carer)
Internal and external contextual factors	Individual characteristics	Knowledge and beliefs about deprescribing- Resident	Resident attitudes and opinion towards deprescribing.	"I think I would have concerns, certainly, with some medication you have to be careful when you come off with the after-effects. And it has to be done in carefully? I wouldn't just accept everything, you have an understanding why." (Care home 1, resident)
		Knowledge and beliefs about deprescribing- Care home staff	Care home staff attitudes and opinion towards deprescribing.	"And they don't necessarily need it, and they come up with it, and we've trialled them coming off of it and they're absolutely fine. And it's nicer for them not being pumped full of medication, rattling around daily. If it's not needed, and they've come here to live out the rest of their life, who really wants to be taking lots of medication every day?" (Care home 12, Supervisor)
		Self-efficacy – Resident	Resident belief in their own knowledge, capabilities and ability to action deprescribing.	"Well, if the medical professional thought it was all right, I would just take their word for it…I think we would take the advice of the doctor." (Care home 1, resident)
		Self-efficacy- Care home staff	Care home staff belief in their own knowledge, capabilities and ability to action deprescribing.	"I think we're able to certainly speak up and challenge. I don't think we'd start questioning and going, they shouldn't be on this, but we would certainly raise concerns and making sure that people are having regular medication reviews, so that they're not on anything unnecessarily." (Care home 4, Deputy manager)
	Outer setting	Cosmopolitanism	The degree to which the care home is networked with other external organisations	"Of course, it's interdependent, because they depend on each other...it depends on the advice of the carer giving to the doctors about what's happening to a patient. And then the doctors summing it up and advising that they should have a certain medication specifically for that patient, or that resident." (Care home 6, resident)

For peer review only

	Inner setting	Access to knowledge and information	Ease of access to digestible information and knowledge about deprescribing and medicines and how to incorporate it into the way staff provide care for residents.	"We just get told, oh, they've had a blood test, they need to have this medication. And it's like, but what did the blood tests show? And it's like they don't always tell us, and it's just like, really? Give us more information, please!" (Care home 9, Deputy manager)
		Available resources	The level of resources the care home dedicated for making changes to the way things are done and on-going operations including physical space and time.	"I think the main thing we need, is time. If I think of the care home I look after, there's 65 residents, so I've got to find… If I want to do it properly, I'd have find at least 65 hours a year to do it regularly, and follow-ups. So it's really quite hard to manage...So time is the biggest problem, and also, and time for care home staff. I mean, they're as pressured as anyone else, for them to actually spend… Take an hour of their day out to talk about each one of their residents, again, is quite onerous on them." (Care home 8, GP)
		Networks and communication	The nature and quality of webs of social networks, and the nature and quality of formal and informal communications within the care home	"Because, obviously, it's on the [Medicine Administration Record] sheet, it's crossed off on our [Medicine Administration Record] sheets, stopped by the GP. It's documented in their care plans. It's documented on our [Patient Centred Software]. It's all well-documented, and like you do medication, you have to follow your [Medicine Administration Record] sheets, so it's always there so everyone can see what's stopped and why, and who stopped it and what date." (Care home 15, Head of Care)
		Tension for change	The degree to which the current situation as intolerable or needing change	" So, yeah, I definitely think that they need to be reviewed regularly, because you don't want people taking medication that they no longer need, that they no longer require, if that makes sense?" (Care home 4, Supervisor)
		Resident needs and resources	The extent to which the needs of care home residents, as well as barriers and facilitators to meet those needs, are accurately known and prioritized by the care home	" So it comes about from us knowing our residents, and being able to make sure we pass that information clearly and accurately and timely onto the surgery, as I mentioned before." (Care home 4, Deputy manager)
	Characteristics of deprescribing (intervention)	Evidence strength and quality	Perceptions of the quality and validity of evidence supporting the belief that deprescribing will have positive outcomes for residents or will do no harm (adverse outcomes).	"I'm aware there is some, but I can't quote anything offhand. We get sent it by our medicines management team quite often, about reviewing. So particularly with antipsychotics...But, yeah, so there is some, and there is our medicines management team at CCG who are really good about supporting us with evidence to things." (Care home 8, GP)
		Cost	Costs of deprescribing and costs associated with implementing deprescribing including investment, supply, and opportunity costs	"I don't think there's any direct cost involved to us, apart from our time. But it would be a nice cost to the resident, because, hopefully, we'd be able to spend more quality time with them, rather than being shove that down your throat, we've got another 40 more to do." (Care home 11, Manager)
		Adaptability	The degree to which deprescribing can be adapted, tailored, refined, or reinvented to meet needs at organisation/provider (care home) and individual (resident) level	"Well, yeah, we're all slightly different. We think we're the same, but we're not and our bodies react differently. But what works - if I come back to this, all these tablets, to my knowledge, work for me." (Care home 14, resident)
		Complexity	Perceived difficulty of the deprescribing	"It's looking for any changes. It's much easier to do this with people who've got capacity, who can tell you. With people with dementia whose communication is limited, you do have to be monitoring more, actually. What are they - are they normal for them? " (Care home 2, Nurse supervisor)
		Relative advantage	Perception of the outcome deprescribing (i.e. better, worse, no change) versus an alternative solution (i.e. continuing medications).	"I don't want her to take things that don't do her - don't do anything." (Care home 2, family carer)
		Trialability	The ability to test deprescribing on a small scale in the care home, and to be able to reverse course (undo implementation) if warranted (i.e. restart medications).	"Well, if it was clear to the medical people, if they wouldn't be, if it was clear to them, and they could go communicate to me, they would try and I'd give it a try, as long as I could go back if I wasn't happy." (Care home 1, resident)

For peer review only

Deprescribing as a complex process

This theme related to the activities and strategies described to implement deprescribing in a care home setting, relating to the CFIR domain of process. Participants discussed deprescribing as a complex process with multiple steps. It included preparation and planning, involvement of multiple people with different roles, and ongoing monitoring and evaluation.

Preparation and planning deprescribing

Conversations about deprescribing could be initiated by a regular medication review, an observed change in the resident, or the resident's preference. Before stopping or reducing medications, care home residents and staff discussed that "*health checks*'' and comprehensive medication reviews should be conducted. Care home staff and the GP expressed how, during a medicine review, there was a need to fully understand the resident's medications and the rationale for each one's use. If a decision was made to deprescribe then the care home needed to know how to do it (i.e. tapering over time or stopping straightaway). Care home staff discussed supporting resident involvement and its difficulties with some residents (e.g., cognitive impairment). They considered that it was important to explain any changes and review how the resident was feeling on certain medications, before stopping or reducing medications.

Engaging multiple people

For deprescribing to happen, all participants discussed how it involved the engagement of various individuals, internal and external to the care homes. Each had a specific role and expertise, which was highlighted by participants. Care home staff, residents and their families expressed how care home staff are residents' advocates and/or intermediaries and often ''*know them best''* (i.e. the resident's individual needs and resources); whereas GPs made the ultimate decisions about if and how deprescribing should

For peer review only

happen. A resident discussed the distinct roles and how they were ''*interdependent because they depend on each other.*'' The involvement of these different people and their respective knowledge and beliefs could help and hinder deprescribing. For example, family members were discussed by care home staff as either encouraging or objecting to the resident's medicines being deprescribed.

The role of a pharmacist in the deprescribing process was more ambiguous. Some residents and care home staff thought the role of a pharmacist was only dispensing medication and, hence, were not involved in any aspect of deprescribing or unsure how they could help. Conversely, if a care home had experienced additional support from pharmacists, such as leading medicine reviews or as a resource for questions about medicines, they were more likely to report how a pharmacist's input was valuable to deprescribing. Pharmacists were considered experts in medicines, but the ultimate decision is made by the GP unless the resident had secondary care where another clinician makes prescribing decisions. Despite the knowledge and expertise of care home staff about the resident and the pharmacist about medicines, all participants (especially, residents) expressed how deprescribing decisions would be done by the GP. Occasionally, other healthcare professionals (e.g., the mental health team, geriatricians, or specific condition nurses) were mentioned but they were often described as being consulted by the GP to make their decisions.

Monitoring and evaluation

Another key part of the deprescribing process that participants discussed was the monitoring and evaluation after medication changes. This was a consistent theme across all participant groups but especially the care home staff as they were the ones observing residents following medication changes. Care home staff emphasised how important it was for them to know about medications to make relevant observations regarding any benefits or side effects. The types of observations required depend on which medications were changed

For peer review only

(e.g., blood pressure, behaviour, alertness, and appetite). The timings and workload required for feedback also varied depending on the complexity of the resident’s health and the types of medication changes. Care home staff and the GP discussed how the effects of stopping or reducing certain medications may take longer than others, thus impacting the monitoring, feedback, and review process. This monitoring was described as actively ongoing to optimise medications for residents and included the possibility of re-starting medications.

Internal and external contextual factors

This theme concerns the contextual factors, including beliefs, abilities, and relationships, reported to influence deprescribing in a care home setting. These CFIR determinants are related to the characteristics of staff and residents (views about deprescribing benefits, ability to question and contribute) as well as the relationships and communication between care homes and the healthcare community (e.g., GPs, pharmacists, and hospitals).

Views about deprescribing and its benefits

Most participants, especially care home staff, reported having mostly positive views and experiences of deprescribing. Often, staff and residents articulated that if medicines were not needed or did not improve the resident's well-being then they would be happy to support deprescribing. Some residents expressed a few concerns because some medicines were perceived as indispensable. Residents discussed concerns about the consequences if these were deprescribed and how some medicines were necessary or too important (for example, antipsychotics for schizophrenia). Care home staff reported the various potential benefits to resident health, quality of life, costs, time, and safety. All participants thought that a deprescribing process should be individualised to the resident and, therefore, adaptable, pilotable, and reversible. These beliefs and attitudes of individuals seem to instil a dynamic, tailored, responsive deprescribing culture in the care home.

For peer review only

279 *Ability to question and contribute*

280 A key characteristic discussed by care home staff and residents was the capacity and confidence to question or raise concerns about medicines with GPs or other prescribers. However, most care home staff and residents felt that they would default to GP advice. Care home staff stated how they did not have the training to question prescribing decisions, but they conveyed that questioning medicines was important to advocate for residents. For those staff who did feel confident to question or raise concerns, the expectation was that they would be listened to by the doctor. Care home staff recognised their knowledge about residents and wanted it to be valued in deprescribing conversations and decisions.

288 *Relationships and communication*

289 Care home staff discussed how mutual trust and respect between care homes and healthcare staff enabled collaboration and good working relationships. The collaboration between GPs and care homes was considered a crucial determinant for deprescribing, despite the support of other healthcare professionals (e.g., pharmacists and other specialists). Supporting the deprescribing process and this collaboration was a good working relationship and information sharing between primary care and the care home. Care homes relied on professionals to review medications, deprescribe, and provide information about monitoring following changes; while the GP relied on the feedback provided by the care home on resident health. The exchanges and information sharing between care home staff and GPs (and other healthcare professionals) determined the access to knowledge and evidence to facilitate deprescribing. This communication was considered an essential component of the deprescribing process. For example, care home staff, the GP and residents discussed how deprescribing could be hindered if medication changes were not communicated to the care home. Care home staff also discussed their role in keeping family members informed, which

For peer review only

was described as more challenging when they were unable to visit homes during the pandemic.

Notably, the distinct types of care home providers (independently owned and part of a larger organisation) did not seem a crucial factor influencing deprescribing. More critical were the working relationships with GP practices and support at individual care homes (such as regular medication reviews or access to pharmacists). The local quality of the relationships and how they worked with healthcare professionals could vary greatly among the providers and individual care homes.

Discussion

The overall aim of this qualitative study was to identify factors which influence deprescribing in English care home settings. The major themes found related to how deprescribing was a complex process, with multiple steps and the involvement of multiple people with distinct roles, and internal and external contextual factors, concerning the characteristics of staff and residents and communication and relationships with the wider healthcare community. Local context and available support were found to be crucial factors across types of care home providers.

The findings add to previous deprescribing research, which described the barriers and enablers (e.g., 21, 22-27, 29); this study described in detail the process of deprescribing and the contextual factors which influence it in the English care home setting. It also considered the various roles and relationships of these different individuals involved in deprescribing. The present study found that how these different people worked together was an important determinant of deprescribing. Participants discussed and stressed how important collaboration and working relationships between care homes and healthcare providers (e.g., GPs and other prescribers) more than the organisational structure of the care home provider. This finding

For peer review only

suggests that these roles and relationships need to be addressed in any successful deprescribing approach in care homes.

It must be recognised that there are power differentials and a ‘hierarchy’ (with GPs holding the decision-making power) in these relationships, and the management of care home residents’ medicines has to take into consideration who has decision-making power (30). Hierarchies and power differentials can affect whose voice is heard; it is well-documented that when power differentials between health and social care settings are present healthcare priorities invariably dominate (39). Fostering collective purpose and identity across sectors could ensure communication and collaboration are not negatively affected (40). Care home residents, family members, care home staff and relevant healthcare professionals should be involved in the deprescribing process (if possible and appropriate) and their respective knowledge recognised and valued for shared decision-making. Additional training, tools, support, and opportunities may need to be made available to care home staff so they can feel confident and able to question or raise concerns when they think that something isn't quite right with the medication. Known enablers of psychological safety and patient safety culture in healthcare teams (such as, professional responsibility, open communication, peer support change-oriented leadership, and learning orientation) could be implemented (41, 42). Future research could examine these separate roles and working relationships as well as resources to support them in greater detail to determine what activities and strategies encourage appropriate deprescribing.

Previous research has recommended nurse champions deprescribing in long-term care facilities (30), but UK care homes do not always have access to qualified nurses. A recent trial introduced pharmacists to lead deprescribing in UK care homes (43), but similar to this study’s findings, the role of the pharmacist was not always understood by care home staff. Any initiative will need to educate not only those individuals selected to champion

For peer review only

deprescribing but also the rest of those involved so there is a shared understanding of each other's role and contribution. The involvement of multiple individuals and organisations also raises issues related to legal concerns, higher workloads, and duty of care (21). Successful integrated working requires trust between health professionals and a clear understanding of responsibilities (44). Further work may need to explore how these are established and negotiated in the care home-primary care working relationships.

The findings support previous work and policy promoting the involvement of multidisciplinary teams (30) and collaboration across care settings (45) to facilitate and implement deprescribing safely and efficiently. Recent policy changes in England set out standards for primary care services to support the delivery of the 'Enhanced Health in Care Homes′ framework (46). A major component of enhanced primary care support includes routine structured medication reviews. This mandated support from primary care is not only an impetus for medicine reviews and potential deprescribing in care homes but also provides regular opportunities for planning, decision-making, and reflecting on their practice. But, evidence suggests that the adoption of the Enhanced Health in Care Homes Framework is not universal or uniform (47). Accordingly, not all care homes will have access to the same support for deprescribing so approaches may need to be tailored to the availability of local resources and, as this study found, the resources and relationships for individual care homes were key factors influencing deprescribing. The findings contribute to the medicine optimisation national priority (18, 48, 49) and NICE recommendations for deprescribing in care homes (17, 18). With an increasing ageing population and greater demand for care in the community, medicine optimisation will continue to be a priority for healthcare services.

Strengths and limitations

A strength of this work is the use of a comprehensive, well-recognised implementation science framework, CFIR, to investigate how to implement deprescribing in

For peer review only

real-world settings (20, 50), specifically in care homes (51). The project demonstrates how CFIR can be used to identify the factors influencing deprescribing into practice (32, 33). The present study findings denoted several domains and constructs of CFIR and the relationships between these determinants. Using this framework can identify crucial determinants, address barriers to deprescribing and help ensure that interventions are effective and sustainable (33, 52). Another strength of the study was the number of respondents that participated in the context and conditions of the pandemic and the high demand for healthcare services where care staff have been so overwhelmed by pressures. Finally, this research adopted a strong patient and public involvement and partnership approach which contributed greatly to the delivery and success of the study.

Some limitations of the study must be acknowledged. Due to the COVID-19 pandemic, care home observations and focus groups were not conducted, which had been originally planned (34); this could have provided a better understanding of everyday practices and insight into experiences than interviews alone. Future work could adopt an ethnographic approach to explore the culture of deprescribing in care homes and observe deprescribing conversations and behaviours. By only conducting interviews, we had to exclude participants who are unable to converse in English and show signs of severe cognitive impairment. These individuals may have different experiences and perspectives. Interviews were with family and friends of these older adults to try to capture these experiences and views. Other limitations of the study were that two-thirds of the sample were care home staff (especially, those in senior positions) and most of the participants were female and white so the findings reflect mostly their perspectives and experiences. Moreover, there may be bias in the sample as regional managers and directors were involved in the selection of participants. Participants were informed that their responses would be kept confidential, and their employment or care (or family member’s care) would not be affected by their responses; they did provide both

For peer review only

positive and negative experiences. There was only one prescriber interviewed, but as we were principally interested in how deprescribing would work in a care home setting, the recruitment of care home staff, residents and families were prioritised. Most previous research has studied the views of prescribers (e.g., 22, 23, 24) and rarely included the views of care home staff.

From this study sample, a difference in views between provider types was not noted but it does not mean there one is not present. Further research could explore the views of more providers and care homes to explore possible differences. Involving care homes in research is challenging and there are several barriers to research participation (53). The COVID-19 pandemic has shown how crucial care homes and their staff are in the care of the frailest and vulnerable. The skills, dedication, and compassion of care home staff not only shape care but can greatly contribute to research.

For peer review only

Conclusion

For deprescribing to be successfully undertaken in care homes, several influencing factors (such as individuals' beliefs and abilities, relationships, and communication) need to be considered and the process itself of deprescribing (including preparation, engaging stakeholders and monitoring). Further work is warranted to design and implement a deprescribing approach for care homes that can be achieved within current structures and resources.

Acknowledgements

We appreciate the feedback from care home staff and residents who attended the patient and public involvement events and who took part in the study. We gratefully acknowledge the input of Donald Nigel Reed.

426 The South West Clinical Research Network (CRN) community support team collaborated and supported the research team.

428 Ethics approval

429 Ethical approval to conduct the study was given by Social Care Research Ethics Committee (19/IEC08/0058). Participants were required to sign a consent form to indicate their willingness to participate. Voluntary participation and the right to ask any questions and to decline participation at any time will be emphasised during the data collection.

433 Funding

434 This project is funded by the National Institute for Health Research (Research for Patient Benefit, Understanding stakeholders' perspectives on implementing deprescribing in care homes (STOPPING), PB-PG-0418-20026). This is independent research supported by the National Institute for Health Research (NIHR) Applied Research Collaboration for the East of England and South West Peninsula. The views expressed in this publication are those of the authors and not necessarily those of the NHS, NIHR, or the Department of Health and Social Care.

441 Authors' contributions

442 KW led the planning, conduct and reporting of the work in this manuscript. KW, JD, EC, GC, LP and KS supported the conceptualisation of the work and acquisition of the funding. TC and GC supported the recruitment of participants and data collection. JR, JD, and AA contributed to the analysis of the data. LP and BW reviewed for important intellectual content. All authors have reviewed and approved the final version of the paper.

447 Competing interests

448 The authors declare that they have no competing interests.

For peer review only

449 **Data sharing statement**

The datasets used and/or analysed during the current study are available from the corresponding author on reasonable request.

For peer review only

References

1. Masnoon N, Shakib S, Kalisch-Ellett L, Caughey GE. What is polypharmacy? A systematic review of definitions. BMC Geriatrics. 2017;17:230.

2. Department of Health and Social Care. Good for you, good for us, good for everybody: A plan to reduce overprescribing to make patient care better and safer, support the NHS, and reduce carbon emissions. 2021.

3. Izza MAD, Lunt E, Gordon AL, Gladman JRF, Armstrong S, Logan P. Polypharmacy, benzodiazepines, and antidepressants, but not antipsychotics, are associated with increased falls risk in UK care home residents: a prospective multi-centre study. European Geriatric Medicine. 2020;11(6):1043-50.

4. Duerden M, Avery T, Payne R. Polypharmacy and medicines optimisation: making it safe and sound. London: The King's Fund; 2013.

5. Kongkaew C, Noyce PR, Ashcroft DM. Hospital admissions associated with adverse drug reactions: a systematic review of prospective observational studies. Annals of Pharmacotherapy. 2008;42(7-8):1017-25.

6. Jyrkka J, Enlund H, Korhonen MJ, Sulkava R, Hartikainen S. Polypharmacy status as an indicator of mortality in an elderly population. Drugs Aging. 2009;26(12):1039-48.

7. Gnjidic D, Hilmer SN, Blyth FM, Naganathan V, Waite L, Seibel MJ, et al. Polypharmacy cutoff and outcomes: five or more medicines were used to identify community-dwelling older men at risk of different adverse outcomes. Journal of clinical epidemiology. 2012;65(9):989-95.

8. Turner JP, Jamsen KM, Shakib S, Singhal N, Prowse R, Bell JS. Polypharmacy cut-points in older people with cancer: how many medications are too many? Supportive care in cancer : official journal of the Multinational Association of Supportive Care in Cancer. 2016;24(4):1831-40.

For peer review only

9. Morin L, Laroche M-L, Texier G, Johnell K. Prevalence of potentially inappropriate medication use in older adults living in nursing homes: a systematic review. Journal of the American Medical Directors Association. 2016;17(9):e861-2. e9.

10. Gnjidic D, Tinetti M, Allore HG. Assessing medication burden and polypharmacy: finding the perfect measure. Expert review of clinical pharmacology. 2017;10(4):345-7.

11. Reeve E, Gnjidic D, Long J, Hilmer S. A systematic review of the emerging definition of ‘deprescribing’ with network analysis: implications for future research and clinical practice. British Journal of Clinical Pharmacology. 2015;80(6):1254-68.

12. Anderson LJ, Schnipper JL, Nuckols TK, Shane R, Sarkisian C, Le MM, et al. A systematic overview of systematic reviews evaluating interventions addressing polypharmacy. American Journal of Health-System Pharmacy. 2019;76(21):1777-87.

13. Iyer S, Naganathan V, McLachlan AJ, Le Conteur DG. Medication Withdrawal Trials in People Aged 65 Years and Older. Drugs & Aging. 2008;25(12):1021-31.

14. Kua C-H, Mak VSL, Huey Lee SW. Health Outcomes of Deprescribing Interventions Among Older Residents in Nursing Homes: A Systematic Review and Meta-analysis. Journal of the American Medical Directors Association. 2019;20(3):362-72.e11.

15. Cooper JA, Cadogan CA, Patterson SM, Kerse N, Bradley MC, Ryan C, et al. Interventions to improve the appropriate use of polypharmacy in older people: a Cochrane systematic review. BMJ Open. 2015;5(12).

16. National Institute for Health and Care Excellence (NICE). Multimorbidity: clinical assessment and management (NG56). 2016.

17. National Institute for Health and Care Excellence (NICE). Medicines optimisation: the safe and effective use of medicines to enable the best possible outcomes (NG5). 2015.

For peer review only

18. National Institute for Health and Care Excellence (NICE). 2017. Available from: https://www.nice.org.uk/advice/ktt18/resources/multimorbidity-and-polypharmacy-pdf-58757959453381.

19. Wang J, Shen JY, Conwell Y, Podsiadly EJ, Caprio TV, Nathan K, et al. Implementation considerations of deprescribing interventions: A scoping review. J Intern Med. 2022.

20. Reeve E, Thompson W, Farrell B. Deprescribing: A narrative review of the evidence and practical recommendations for recognizing opportunities and taking action. European journal of internal medicine. 2017;38:3-11.

21. Palagyi A, Keay L, Harper J, Potter J, Lindley RI. Barricades and brickwalls–a qualitative study exploring perceptions of medication use and deprescribing in long-term care. BMC geriatrics. 2016;16(1):15.

22. Schuling J, Gebben H, Veehof LJG, Haaijer-Ruskamp FM. Deprescribing medication in very elderly patients with multimorbidity: the view of Dutch GPs. A qualitative study. BMC family practice. 2012;13(1):56.

23. Moen J, Norrgård S, Antonov K, Nilsson JLG, Ring L. GPs' perceptions of multiple-medicine use in older patients. Journal of evaluation in clinical practice. 2010;16(1):69-75.

24. Anthierens S, Tansens A, Petrovic M, Christiaens T. Qualitative insights into general practitioners views on polypharmacy. BMC family practice. 2010;11(1):65.

25. Linsky A, Simon SR, Bokhour B. Patient perceptions of proactive medication discontinuation. Patient Education and Counseling. 2015;98(2):220-5.

26. Linsky A, Simon SR, Marcello TB, Bokhour B. Clinical provider perceptions of proactive medication discontinuation. Am J Manag Care. 2015;21(4):277-83.

For peer review only

27. Kalogianis MJ, Wimmer BC, Turner JP, Tan ECK, Emery T, Robson L, et al. Are residents of aged care facilities willing to have their medications deprescribed? Research in Social and Administrative Pharmacy. 2016;12(5):784-8.

28. Lo SY, Reeve E, Page AT, Zaidi STR, Hilmer SN, Etherton-Beer C, et al. Attitudes to Drug Use in Residential Aged Care Facilities: A Cross-Sectional Survey of Nurses and Care Staff. Drugs & Aging. 2021;38(8):697-711.

29. Turner JP, Edwards S, Stanners M, Shakib S, Bell JS. What factors are important for deprescribing in Australian long-term care facilities? Perspectives of residents and health professionals. BMJ Open. 2016;6(3):e009781.

30. Heinrich CH, Hurley E, McCarthy S, McHugh S, Donovan MD. Barriers and enablers to deprescribing in long-term care facilities: a 'best-fit' framework synthesis of the qualitative evidence. Age and Ageing. 2022;51(1).

31. Page AT, Clifford RM, Potter K, Schwartz D, Etherton-Beer CD. The feasibility and effect of deprescribing in older adults on mortality and health: a systematic review and meta-analysis. British Journal of Clinical Pharmacology. 2016;82(3):583-623.

32. Ailabouni NJ, Reeve E, Helfrich CD, Hilmer SN, Wagenaar BH. Leveraging implementation science to increase the translation of deprescribing evidence into practice. Research in Social and Administrative Pharmacy. 2022;18(3):2550-5.

33. Ronquillo C, Day J, Warmoth K, Britten N, Stein K, Lang I. An Implementation Science Perspective on Deprescribing. Public Policy & Aging Report. 2018;28(4):134-9.

34. Warmoth K, Day J, Cockcroft E, Reed DN, Pollock L, Coxon G, et al. Understanding stakeholders' perspectives on implementing deprescribing for older people living in long-term residential care homes: the STOPPING study protocol. Implementation Science Communications. 2020;1(1).

For peer review only

35. Damschroder LJ, Aron DC, Keith RE, Kirsh SR, Alexander JA, Lowery JC. Fostering implementation of health services research findings into practice: a consolidated framework for advancing implementation science. Implementation science. 2009;4(1):50.

36. Greenhalgh T, Robert G, Macfarlane F, Bate P, Kyriakidou O. Diffusion of innovations in service organizations: systematic review and recommendations. The Milbank Quarterly. 2004;82(4):581-629.

37. Guest G, Bunce A, Johnson L. How Many Interviews Are Enough? An Experiment with Data Saturation and Variability. Field Methods. 2006;18(1):59–82

38. Ritchie J, Lewis J, McNaughton Nicholls C, Ormston R. Qualitative research practice: A guide for social science students and researchers. 2nd ed. London: SAGE 2014

39. Gordon AL, Spilsbury K, Achterberg WP, Adams R, Jones L, Goodman C. From Warkworth House to the 21st century care homes: progress marked by persistent challenges. Age and Ageing. 2022;51(7).

40. Amador S, Goodman C, Mathie E, Nicholson C. Evaluation of an Organisational Intervention to Promote Integrated Working between Health Services and Care Homes in the Delivery of End-of-Life Care for People with Dementia: Understanding the Change Process Using a Social Identity Approach. Int J Integr Care. 2016;16(2):14.

41. O'donovan R, McAuliffe E. A systematic review of factors that enable psychological safety in healthcare teams. International Journal for Quality in Health Care. 2020;32(4):240-50.

42. Battard J. Nonpunitive response to errors fosters a just culture. Nursing Management. 2017;48(1):53-5.

43. Birt L, Dalgarno L, Wright DJ, Alharthi M, Inch J, Spargo M, et al. Process evaluation for the Care Homes Independent Pharmacist Prescriber Study (CHIPPS). BMC Health Services Research. 2021;21(1).

For peer review only

44. Davies SL, Goodman C, Bunn F, Victor C, Dickinson A, Iliffe S, et al. A systematic review of integrated working between care homes and health care services. BMC Health Services Research. 2011;11(1):320.

45. Conklin J, Farrell B, Suleman S. Implementing deprescribing guidelines into frontline practice: Barriers and facilitators. Research in Social and Administrative Pharmacy. 2019;15(6):796-800.

46. NHS England and NHS Improvement. The Framework for Enhanced Health in Care Homes;. In: England N, editor. London, UK2020.

47. Warmoth K, Goodman C. Models of Care and Relationships with Care Homes: Cross-Sectional Survey of English General Practices. International Journal of Environmental Research and Public Health. 2022;19(22):14774.

48. Naylor C, Imison C, Addicott R, Buck D, Goodwin N, Harrison T, et al. Transforming our helath care system: Ten priorities for commissioners. The King's Fund; 2015.

49. NHS England. Regional Medicines Optimisation Committee 2017 [Available from: https://www.england.nhs.uk/publication/regional-mediciens-optimisation-committee-operating-guidance-and-recruitment-information/.

50. Sawan M, Reeve E, Turner J, Todd A, Steinman MA, Petrovic M, et al. A systems approach to identifying the challenges of implementing deprescribing in older adults across different health-care settings and countries: a narrative review. Expert Review of Clinical Pharmacology. 2020;13(3):233-45.

51. Alldred DP, Kennedy M, Hughes C, Chen TF, Miller P. Interventions to optimise prescribing for older people in care homes. Cochrane Database of Systematic Reviews. 2016(2).

For peer review only

52. Gnjidic D, Le Couteur DG, Hilmer SN. Discontinuing drug treatments. BMJ. 2014: 349.

53. Law E, Ashworth R. Facilitators and Barriers to Research Participation in Care Homes: Thematic Analysis of Interviews with Researchers, Staff, Residents and Residents' Families. Journal of Long-Term Care. 2022;0(2022):49.

For peer review only

STOPPING

Understanding stakeholders' perspectives on implementing deprescribing in care homes (STOPPING)

TOPIC GUIDE WITH CARE HOME STAFF AND HEALTHCARE PROFESSONALS

Introduction

We are here today to talk about your experiences with and views of deprescribing (stopping a medication or reducing its dosage) in care homes. The purpose is to get your perceptions of how reducing or stopping some medicines would work in a care home setting so we can identify the key barriers, facilitators and contextual factors influencing it. I am not here to share information, or to give you my opinions. Your perceptions are what matter. There are no right or wrong or desirable or undesirable answers. You can disagree with each other, and you can change your mind. I would like you to feel comfortable saying what you really think and how you really feel.

As you know everything is confidential.

Topics

Knowledge & beliefs about the intervention

What do you think about reducing or stopping some medicines for people living in care homes?

Probes. Why do you think that? Do you have any concerns? Are there any benefits?

Do you think reducing or stopping some medicines will be beneficial in your care home?

Probes. Why or Why not? Do you have any concerns?

Relative advantage

Do you think are the advantages of reducing or stopping some medicines for people living in care homes compared to continuing their medicines?

Probes. Why? Is that your experience? Anything else?

Do you think are the disadvantages of reducing or stopping some medicines for people living in care homes compared to continuing their medicines?

Probes. Why? Is that your experience? Anything else?

Tension for change

Is there a strong need for reducing or stopping some medicines?

Probes. Why or why not? Do others see a need for it?

How essential is this reducing or stopping some medicines to meet the needs of the individuals served by your care home?

How do you feel about current programs/practices/process that are available?

Probes. What do they do well? To what extent do current programs fail to meet existing needs?

Complexity

How complicated is it to reduce or stop some medicines for care home residents?

Probes. Please consider the following aspects: duration, scope, intricacy and number of steps involved

For peer review only

STOPPING

Trialability

What do you think about trying to stop or reduce a medicine (or medicines) for a short period?

Probes. What would that be like? Would it be helpful? Harmful? Why or why not?

Evidence strength & quality

What kind of information or evidence are you aware of that shows whether or not the reducing or stopping some medicines will work in your care home?

Probes. What evidence have you heard about? Practice guidelines? Published literature? Co-workers? Other care homes? How does this knowledge affect your view?

What kind of supporting evidence or proof is needed about the effectiveness of reducing or stopping some medicines to get staff on board?

Probes. Co-workers? Administrative leaders?

Planning

What needs to be done beforehand so that reducing or stopping medicines can happen in care homes?

Probes. Why? Is that your experience? What steps should be included? Anything else? What is your role in the planning?

Engaging

What do you think the role of the care home and its staff should be in reducing and stopping some medicines?

Probes. How would that like look? To what extent?

What do you think the role of the GPs should be?

Probes. How would that like look? To what extent?

What do you think the role of the pharmacist should be?

Probes. How would that like look? To what extent?

Is there anyone else who should be involved?

Probes. How would that like look? To what extent?

Reflecting & Evaluating

What do you think should be the outcome of reducing or stopping some medicines for people living in care homes?

Probes. Why? Is that your experience? Anything else?

What should happen after reducing or stopping some medicines?

Probes. What information should be collected? How often? Feedback given?

Communication & Network

How do you know when there have been changes to the resident's medicines?

Probes. What gets in the way of knowing? Can this be improved? If so how?

For peer review only

STOPPING

Can you describe your working relationships with your colleagues?

Probes. With colleagues in your care home? With colleagues in other care homes? Can you tell me a story about a time you needed to work with others to solve a problem? Or to implement reducing to stopping a resident's medicines?

Do you understand why there have been changes to a resident's medicines?

Probes. Why? What would aid your understanding? What stops you from understanding? How could this be communicated better?

Cosmopolitanism

What kind of information sharing do you have with other professionals and organisations outside your care home, either related to the medicines, or more generally?

Probes. What professional or care home networking do you engage in? Listservs? Local or national conferences? Trainings?

Executing & Personal Attributes

Please tell me about any experiences that you may have with stopping or reducing some of the residents' medicines.

Probes. How did it go? What happened? When been done well? When has it not gone so well? What would help work better in the future? Why was that the case? Was anything done by the care home staff that helped this experience? Or impeded it? What problems or issues did you experience, if any?

Available resources

Do you expect to have sufficient resources to implement and administer reducing or stopping some medicines?

Probes. [If Yes] What resources are you counting on? Are there any other resources that you received, or would have liked to receive? What resources will be easy to procure?
[If no] What resources will not be available?

Cost

What are the costs associated with reducing or stopping a resident's medicines?

Probes. What costs will be incurred? What cost were considered when deciding to implement?

Patient needs & resources

How do the residents' needs and preferences get included in decisions to change their medicines?

Probes. Why were they included or not included? How 'in touch' were the people involved? Were they prioritised over other things? Has anything been altered or changed to meet the resident's needs? What would you like your input to be?

How would you feel about stopping or reducing some of the resident's medicines?

Probes. Is there anything that would prevent you? Why?

Self-efficacy

Do you think you can question the amount of medicines that residents are taking?

For peer review only

STOPPING

Probes. How confident are you to do this? Would you do it? Have you? Why or Why not?

Closure

Is there any other information regarding your experiences or opinions that you think would be useful for me to know?

Thank you very much for meeting with me. Your time is very much appreciated and your comments have been very helpful.

For peer review only

STOPPING

Understanding stakeholders' perspectives on implementing deprescribing in care homes (STOPPING)

INTERVIEW TOPIC GUIDE WITH CARE HOME RESIDENT

Introduction

Good morning/afternoon. My name is Krystal Warmoth. I am going to ask you some questions about reducing or stopping some prescription medicines. For example, someone may need to stop taking a medicine that may no longer be providing benefit or has side effects (like falling) as they get older, or reducing the dose of a medication so that the person doesn't have to take as many tablets each day. We still don't really know how to make it work in real-life settings, like care homes. In this project, we aim to study how reducing or stopping some medicines can be best done in care homes, considering different views and settings.

We are not wanting to change your medicines, we are just wanting to chat with you to understand what happens at the moment, in terms of how your medicines may or may not be stopped or reduced. And discuss your thoughts on this happening, and how we could one day, make sure it's done well.

Again, I want to remind you that your responses will be kept confidential and you do not have to answer any question if you do not wish to. You can also stop the interview at any time.

Before we start, I want to check that you are happy to be interviewed and for the interview to be recorded.

Interview

1. *To start, I want to know what your opinions are about reducing or stopping some medicines. What do you think about the idea of reducing or stopping some medicines?* ***Knowledge & beliefs***

   Probes. Why do you think that? Do you have any concerns? Are there any benefits?

2. *Do you think there are the* advantages of reducing or stopping some medicines*?* ***Knowledge & beliefs & Relative advantage***

   Probe. Why or Why not? Do you think are the disadvantages? Why or Why not?

3. *What do you think about trying to stop or reduce a medicine (or medicines) for a short period of time?* ***Trialability***

   Probes. What would that be like? Who would lead it? Would it be helpful? Harmful? Why or why not?

4. *What things will need to happen when planning to reduce or stop some medicines?* ***Planning***

   Probes. Why? Is that your experience? What steps should be included? Anything else? What is your role in the planning?

[CHECK IF THEY ARE HAPPY TO CONTINUE OR WOULD LIKE TO TAKE A BREAK]

5. *Do you think different approaches to reducing or stopping some medicines would be needed for different people?* ***Adaptability***

   Probes. Why or why not? What kinds of changes do you think would need to be made? Do you think these changes are possible? Why or Why not?

For peer review only

STOPPING

6. *What do you think the role of the care home and its staff should be in reducing and stopping some medicines?* ***Engaging***

   Probes. How would that like look? To what extent?

7. *What do you think the role of the GPs should be in reducing and stopping some medicines?* ***Engaging***

   Probes. How would that like look? To what extent?

8. *What do you think the role of the pharmacist should be in reducing and stopping some medicines?* ***Engaging***

   Probes. How would that like look? To what extent?

9. *Is there anyone else that needs to be involved in how to reduce or stop some medicines?* ***Engaging***

   Probes. Why? How would that like look? To what extent? What about family and friends?

[CHECK IF THEY ARE HAPPY TO CONTINUE OR WOULD LIKE TO TAKE A BREAK]

10. *What do you think should be the impact or end result of reducing or stopping some medicines for people living in care homes?* ***Reflecting & Evaluating***

    Probes. Why? Is that your experience? Anything else? What about quality of life? Side effects?

11. *Should anything else happen after someone's medicines have been reduced or stopped?* ***Reflecting & Evaluating***

    Probes. What information should be collected? How often? Feedback given?

12. *Did you know when there have been changes to your medicines?* ***Communication & Network***

    Probes. How did you know? What gets in the way of knowing?

13. *Did you understand why there were changes to your medicines?* ***Knowledge & beliefs***

    Probes. Why? What would aid your understanding? What stops you from understanding? How could this be communicated better?

[CHECK IF THEY ARE HAPPY TO CONTINUE OR WOULD LIKE TO TAKE A BREAK]

14. *Now, I would like to know about your own experiences with reducing or stopping some medicines. Please tell me about any experiences that you may have with stopping or reducing some of your medicines while you [they] have been living in the care home.* ***Executing & Personal Attributes***

    Probes. How did it go? What happened? When has it been done well? When has it not gone so well? What would help it work better in the future? Why was that the case? Was anything done by the care home staff that helped this experience? Or impeded it? What problems or issues did you experience, if any?

[IF CANNOT REMEMBER AN EXPERIENCE -> SKIP TO QUESTION 16]

[CHECK IF THEY ARE HAPPY TO CONTINUE OR WOULD LIKE TO TAKE A BREAK]

15. *Thinking back to your experience of medicines being stopped or reduced. How did your needs and preferences get included in decisions to change your medicines?* ***Patient needs & resources***

For peer review only

STOPPING

Probes. Why were they included or not included? How 'in touch' were the people involved? Were they prioritised over other things? Has anything been altered or changed to meet your needs? What would you like your input to be?

16. *Do you think you can question the amount of medicines that you are taking?* ***Self-efficacy***

Probes. How confident are you to do this? Would you do it? How would you talk to about this? Have you? Why or Why not?

17. How would you feel about stopping or reducing some of your medicines? ***Patient needs & resources***

Probes. Would you do it? Is there anything that would prevent you doing it? Why?

Closure

Is there any other information regarding your opinions or experiences that you think would be useful for me to know?

Again, I want to let you know that your medicines are not being changed based on what we have talked about. If you wish to change your medications as a result of this discussion, we will let the care home staff know so you can discuss this with your general practitioner and any other relevant people. Please do not change your medicines until you have talked with the appropriate healthcare professional.

Thank you very much for meeting with me. Your time is very much appreciated and your comments have been very helpful.

To finish, I am going to ask a few questions to get some basic information about you.

For peer review only

STOPPING

Understanding stakeholders' perspectives on implementing deprescribing in care homes (STOPPING)

INTERVIEW TOPIC GUIDE WITH FAMILY OR FRIEND

Introduction

Good morning/afternoon. My name is Krystal Warmoth. I am going to ask you some questions about reducing or stopping some prescription medicines. For example, someone may need to stop taking a medicine that may no longer be providing benefit or has side effects (like falling) as they get older, or reducing the dose of a medication so that the person doesn't have to take as many tablets each day. We still don't really know how to make it work in real-life settings, like care homes. In this project, we aim to study how reducing or stopping some medicines can be best done in care homes, considering different views and settings.

We are not wanting to change [INSERT RESIDENT'S NAME]'s medicines, we are just wanting to chat with you to understand what happens at the moment, in terms of how the [INSERT RESIDENT'S NAME]'s medicines may or may not be stopped or reduced. And discuss your thoughts on this happening, and how we could one day, make sure it's done well.

Again, I want to remind you that your responses will be kept confidential and you do not have to answer any question if you do not wish to. You can also stop the interview at any time.

Before we start, I want to check that you are happy to be interviewed and for the interview to be recorded.

Interview

18. *To start, I want to know what your opinions are about reducing or stopping some medicines. What do you think about the idea of reducing or stopping some medicines?* ***Knowledge & beliefs***

    Probes. Why do you think that? Do you have any concerns? Are there any benefits?

19. *Do you think there are the* advantages of reducing or stopping some medicines*?* ***Knowledge & beliefs & Relative advantage***

    Probe. Why or Why not? Do you think are the disadvantages? Why or Why not?

20. *What do you think about trying to stop or reduce a medicine (or medicines) for a short period of time?* ***Trialability***

    Probes. What would that be like? Who would lead it? Would it be helpful? Harmful? Why or why not?

21. *What things will need to happen when planning to reduce or stop some medicines?* ***Planning***

    Probes. Why? Is that your experience? What steps should be included? Anything else? What is your role in the planning?

[CHECK IF THEY ARE HAPPY TO CONTINUE OR WOULD LIKE TO TAKE A BREAK]

22. *Do you think different approaches to reducing or stopping some medicines would be needed for different people living in a care home?* ***Adaptability***

    Probes. Why or why not? What kinds of changes do you think would need to be made? Do you think these changes are possible? Why or Why not?

For peer review only

STOPPING

23. *What do you think the role of the care home and its staff should be in reducing and stopping some medicines?* ***Engaging***

    Probes. How would that like look? To what extent?

24. *What do you think the role of the GPs should be in reducing and stopping some medicines?* ***Engaging***

    Probes. How would that like look? To what extent?

25. *What do you think the role of the pharmacist should be in reducing and stopping some medicines?* ***Engaging***

    Probes. How would that like look? To what extent?

26. *Is there anyone else that needs to be involved in how to reduce or stop some medicines?* ***Engaging***

    Probes. Why? How would that like look? To what extent? What about family and friends?

[CHECK IF THEY ARE HAPPY TO CONTINUE OR WOULD LIKE TO TAKE A BREAK]

27. *What do you think should be the impact or end result of reducing or stopping some medicines for people living in care homes?* ***Reflecting & Evaluating***

    Probes. Why? Is that your experience? Anything else? What about quality of life? Side effects?

28. *Should anything else happen after someone's medicines have been reduced or stopped?* ***Reflecting & Evaluating***

    Probes. What information should be collected? How often? Feedback given?

29. *Did you know when there have been changes to [INSERT RESIDENT'S NAME]'s medicines?* ***Communication & Network***

    Probes. How did you know? What gets in the way of knowing?

30. *Did you understand why there were changes to [INSERT RESIDENT'S NAME]'s medicines?* ***Knowledge & beliefs***

    Probes. Why? What would aid your understanding? What stops you from understanding? How could this be communicated better?

[CHECK IF THEY ARE HAPPY TO CONTINUE OR WOULD LIKE TO TAKE A BREAK]

31. *Now, I would like to know about your own experiences with reducing or stopping some medicines. Please tell me about any experiences that you may have with stopping or reducing some of [INSERT RESIDENT'S NAME]'s medicines while they have been living in the care home.* ***Executing & Personal Attributes***

    Probes. How did it go? What happened? When has it been done well? When has it not gone so well? What would help it work better in the future? Why was that the case? Was anything done by the care home staff that helped this experience? Or impeded it? What problems or issues did you experience, if any?

[IF CANNOT REMEMBER AN EXPERIENCE -> SKIP TO QUESTION 16]

[CHECK IF THEY ARE HAPPY TO CONTINUE OR WOULD LIKE TO TAKE A BREAK]

For peer review only

STOPPING

32. *Thinking back to your experience of medicines being stopped or reduced. How did your needs and preferences get included in decisions to change [INSERT RESIDENT'S NAME]'s medicines? **Patient needs & resources***

    Probes. Why were they included or not included? How 'in touch' were the people involved? Were they prioritised over other things? Has anything been altered or changed to meet your [resident's] needs? What would you like your input to be?

33. *Do you think you can question the amount of medicines that [INSERT RESIDENT'S NAME] are taking? **Self-efficacy***

    Probes. How confident are you to do this? Would you do it? How would you talk to about this? Have you? Why or Why not?

34. How would you feel about stopping or reducing some of *[INSERT RESIDENT'S NAME]'s* medicines? ***Patient needs & resources***

    Probes. Would you do it? Is there anything that would prevent you doing it? Why?

Closure

Is there any other information regarding your opinions or experiences that you think would be useful for me to know?

Again, I want to let you know that your [resident's] medicines are not being changed based on what we have talked about. If you wish to change your [resident's] medications as a result of this discussion, we will let the care home staff know so you can discuss this with your general practitioner and any other relevant people. Please do not change your [resident's] medicines until you have talked with the appropriate healthcare professional.

Thank you very much for meeting with me. Your time is very much appreciated and your comments have been very helpful.

To finish, I am going to ask a few questions to get some basic information about you.

For peer review only

STOPPING CODEBOOK

Characteristics of deprescribing	
Evidence Strength & Quality	Definition: Stakeholders' perceptions of the quality and validity of evidence supporting the belief that deprescribing will have positive outcomes for residents or will do no harm (adverse outcomes).<br><br>Inclusion Criteria: Include statements regarding awareness of evidence that deprescribing will work including the strength and quality of evidence (e.g. NICE guidelines, news articles), as well as the absence of evidence or a desire for different types of evidence.<br><br>Exclusion Criteria: Exclude or double code statements regarding the receipt of evidence as an engagement strategy to *Process, Key Stakeholders*. For example, statements regarding what evidence is needed for engagement.
Relative Advantage	Definition: Stakeholders' perception of the outcome deprescribing (i.e. better, worse, no change) versus an alternative solution (i.e. continuing medications). Split into **organisation level** (care home staff) and **individual level** (residents and families) including statements on the advantages and disadvantages of deprescribing.<br><br>Inclusion Criteria: Include statements that demonstrate the deprescribing is better (or worse/the same) than not changing medication.<br><br>Exclusion Criteria: Exclude statements that demonstrate a strong need for deprescribing and/or that the current situation is untenable and code to *Tension for Change*.
Adaptability	Definition: The degree to which deprescribing can be adapted, tailored, refined, or reinvented to meet needs at **organisation/provider** (care home) and **individual** (resident) level.<br><br>Inclusion Criteria: At **organisation level,** include statements regarding the (in)ability to adapt deprescribing to care home context e.g., perceptions on whether a different approach is needed. **Individual level** determinants may include statements on flexible, holistic and person-centered approaches.
Trialability	Definition: The ability to test deprescribing on a small scale in the care home, and to be able to reverse course (undo implementation) if warranted (i.e. restart medications).<br><br>Inclusion Criteria: At **organisation level**, include statements related to whether the care home piloted deprescribing for a short period of time (in the past or has plans to in the future) and comments about whether they believe it is (im)possible to stop medications for a short period of time. At **individual level**, include statements about resident's views on stopping medications for a short time.

For peer review only

Complexity	Definition: Perceived difficulty of the deprescribing. On an **organisation/provider** level, this is reflected by duration, scope, radicalness, disruptiveness, centrality, and intricacy and number of steps required to implement. On an **individual** level this is reflected by individual complexities such as severity of health condition or cognitive abilities.<br><br>Inclusion Criteria: Code statements regarding the complexity of the deprescribing process.<br><br>Exclusion Criteria: Exclude statements regarding the complexity of implementation and code to the appropriate CFIR code, e.g., difficulties related to space are coded to *Inner setting, Available Resources* and difficulties related to engaging participants in deprescribing are coded to *Process, Innovation Participants*.
Design Quality & Packaging	Definition: Perceived excellence in how the deprescribing tool, guidance or approach is bundled, presented, and assembled.<br><br>Inclusion Criteria: Include statements regarding the quality of the materials and packaging.<br><br>Exclusion Criteria: Exclude statements regarding the presence or absence of materials and code to *Available Resources*.<br>Exclude statements regarding the receipt of materials as an engagement strategy and code to *Engaging*
Cost	Definition: Costs of deprescribing and costs associated with implementing deprescribing including investment, supply, and opportunity costs.<br><br>Inclusion Criteria: Include statements related to the cost of deprescribing and its implementation. Code statements related to medication use including waste (implied cost).<br><br>Exclusion Criteria: Exclude statements related to physical space and time, and code to *Available Resources*. In a research study, exclude statements related to costs of conducting the research components (e.g., funding for research staff, participant incentives).
**Outer Setting**	
Cosmopolitanism	Definition: The degree to which the care home is networked with other external organisation i.e. primary care networks (**community/systems** level) and family carers.<br><br>Inclusion Criteria: At a **community/systems level**, include descriptions of outside group memberships and networking done outside the care home. For example, communication between the care homes with GPs and pharmacists. At an **individual level**, include statements about communication between family carer, GP and care home, also double-code to innovation participants.

For peer review only

	Exclusion Criteria: Exclude statements relating to information sharing or networking internally code to *Inner setting, Networks & Communication.*<br>Exclude statements relating to stakeholder role in deprescribing and code in *Process, external change agents/key stakeholders*.
External Policy & Incentives	Definition: A broad construct that includes external strategies to spread innovations including policy and regulations (governmental or other central entity), external mandates, recommendations and guidelines, pay-for-performance, collaboratives, and public or benchmark reporting.<br><br>Inclusion Criteria: Include descriptions of external performance measures from the system. For example, requirements for annual medication reviews (Quality Outcome frameworks).
**Inner Setting**	
Resident Needs & Resources	Definition: The extent to which the needs of care home residents, as well as barriers and facilitators to meet those needs, are accurately known and prioritized by the care home (**organisation/provider** level).<br><br>Inclusion Criteria: Include statements demonstrating (lack of) awareness of the needs and resources of care home residents. Analysts may be able to infer the level of awareness based on statements about: 1. Perceived need for deprescribing based on care home residents and if deprescribing will meet those needs; 2. Barriers and facilitators of care home residents to participating in deprescribing.<br><br>Exclusion Criteria: Exclude statements that demonstrate a strong need for the innovation and/or that the current situation is untenable and code to *Inner setting*, *Tension for Change*.<br>Double code statements relating to resident and family perception of need for deprescribing (**individual** level) to *Process, Innovation participants.*
Networks & Communications	Definition: The nature and quality of webs of social networks, and the nature and quality of formal and informal communications within the care home (**organisation/provider** level).<br><br>Inclusion Criteria: Include statements about general networking, communication, and relationships in the care home, such as descriptions of meetings, email groups, or other methods of keeping people connected and informed, and statements related to working relationships. Includes how care home staff know when and why medication has been changed (or not).<br><br>Exclusion Criteria: Exclude statements related to knowledge and information regarding deprescribing and code to *Access to*

For peer review only

	Knowledge & Information. Exclude statements related to stakeholder's (care home staff) role in implementation and code to *Process, Key Stakeholders.* Exclude descriptions of external group memberships and networking done outside the organisation and code to *Outer setting, Cosmopolitanism.*
Tension for Change	Definition: The degree to which stakeholders perceive the current situation as intolerable or needing change.<br><br>Inclusion Criteria: Include statements that (do not) demonstrate a strong need for deprescribing and/or that the current situation is untenable. For example, statements acknowledging residents are on 'too many' medication and suggest 'need' to change/deprescribe.<br><br>Exclusion Criteria: Exclude statements regarding specific needs of individuals that demonstrate a need for deprescribing, but do not necessarily represent a strong need or an untenable status quo, and code to *Inner setting, Resident Needs and Resources.*
Available Resources	Definition: The level of resources the care home (**organisation/provider** level) dedicated for making changes to the way things are done and on-going operations including physical space and time.<br><br>Inclusion Criteria: Include statements related to the presence or absence of sufficient resources specific to deprescribing that is being implemented.<br><br>Exclusion criteria: At a **community/system** level, resource changes/challenges as a result of COVID-19 double-coded as *Outer Setting, External Policy*. For example, reduced in-person medication reviews (impacting **organisation/provider** and **individual** levels).
Access to Knowledge & Information	Definition: Ease of access to digestible information and knowledge about deprescribing and how to incorporate it into the way staff provide care for residents.<br><br>Inclusion Criteria: Include statements related to care home staff access to knowledge and information regarding deprescribing (**organisation/provider**).
Compatibility	Definition: The degree of tangible fit between meaning and values attached to deprescribing by involved individuals, how those align with individuals' own norms, values, and perceived risks and needs, and how the deprescribing (including tools, guidance and approaches) fits with existing workflows and systems.<br><br>Inclusion Criteria: Include statements that demonstrate the level of compatibility deprescribing has with organizational values and work processes. Include statements that the innovation did or did not need to be adapted.<br><br>Exclusion Criteria

For peer review only

	Exclude or double code statements regarding the priority of deprescribing on compatibility with organizational values to *Relative Priority* e.g., if an deprescribing is not prioritized because it is not compatible with organizational values.
**Individual characteristics (staff and resident determinants)**	
Knowledge & Beliefs	Definition: Individuals' attitudes toward and value placed on the deprescribing, as well as familiarity with facts, truths, and principles related to deprescribing.<br><br>Inclusion Criteria: Include statements from care home staff (**organisation/provider**), residents and families (**individual**) about their attitudes and opinions about deprescribing. For example, statements about generational differences in accepting opinion of doctor regarding medication.<br><br>Exclusion Criteria: code statements related to stopping medication for a short time to *Intervention Characteristics, Trialability*. Double code knowledge of prescriptions (rationale, benefits) to *Inner setting>Resident needs and resources*.
Self-efficacy	Definition: Individual belief in their own knowledge, capabilities, and ability to action deprescribing.<br><br>Inclusion Criteria: Includes statements related to ability to question amount of medication on both an **organisation/provider** (care home staff) and **individual** (residents and families) level. Not only cognitive aspects (i.e. beliefs in own ability to question prescriptions) but also practical aspects (i.e. staff role restrictions).<br><br>Exclusion criteria: Double code information about staff role restrictions to *Process>Key Stakeholders* or *Outer Setting>External Policy*
**Process**	
Planning	Definition: The degree to which a scheme or method of behavior and tasks for implementing deprescribing are developed in advance, and the quality of those schemes or methods.<br><br>Inclusion Criteria: Include evidence of pre-implementation diagnostic assessments and planning (i.e. what should be done before deprescribing). For example, medication review, physical health checks. Include the perspectives of care home staff (**organisation/provider**) and residents (**individual**).
External Change Agents	Definition: Individuals who are affiliated with *outside* care home stakeholders who formally influence or facilitate deprescribing. These include GPs and pharmacists (**community/system** level).<br><br>Inclusion Criteria: Include statements related to how the external change agent (e.g. primary care networks) became engaged with

For peer review only

	deprescribing and what their *role* is in implementation. For example, their capabilities, motivation, and skills.<br><br>Exclusion Criteria: Note: It is important to clearly define what roles are external and internal to the organisation. If staff member *internal* to the organisation, it should be coded elsewhere (*Key stakeholders*) even though their support may overlap with what would be expected from an External Change Agent. Although family members are external to the organisation, information about role should be coded to Innovation Participants due to their position in advocating for the health of the resident/their relative.
Key Stakeholders	Definition: Care home staff role from *within* the care home (**organisation/provider** level) that are directly impacted by deprescribing.<br><br>Inclusion Criteria: Include statements related to how care home staff became engaged with deprescribing and what their role is in implementation. For example, staff perception of their role in challenging resident prescriptions, requesting reviews, initiating deprescribing discussions, reporting drug errors (professional fear of malpractice).<br><br>Exclusion Criteria: Exclude statements about general networking, communication, and relationships in the organisation, such as descriptions of meetings, email groups, or other methods of keeping people connected and informed, and statements related to team formation, quality, and functioning, and code to *Inner setting, Networks & Communications.* If statements relate to belief in capabilities rather than practical roles then code to *Individual Characteristics, Self-efficacy.*
Innovation Participants	Definition: Care home resident role in deprescribing (**individual** level). Links with family carers.<br><br>Inclusion Criteria: Include statements that capture whether or not resident needs and preferences are included in decisions around deprescribing, how residents are engaged/involved with deprescribing, and difficulties related to engaging residents in deprescribing.<br><br>Exclusion Criteria: Exclude statements demonstrating (lack of) awareness of the needs and resources of those served by the organisation and code to *Inner setting, Resident Needs & Resources> Organisation level.*
Reflecting & Evaluating	Definition: Quantitative and qualitative feedback about the progress and quality of deprescribing. Includes statements about what should happen after or be the outcome of deprescribing.

	Inclusion Criteria: Include statements that refer to the care homes (lack of) assessment of the impact and outcomes related to deprescribing. For example, how deprescribing can be done better. When participants discuss the need for monitoring following deprescribing.<br><br>Exclusion Criteria: Exclude statements that capture reflecting and evaluating that participants may do during the interview, for example, related to the success of the implementation, and code to *Individual Characteristics, Knowledge & Beliefs (about deprescribing)*.

For peer review only

COREQ (COnsolidated criteria for REporting Qualitative research) Checklist

A checklist of items that should be included in reports of qualitative research. You must report the page number in your manuscript where you consider each of the items listed in this checklist. If you have not included this information, either revise your manuscript accordingly before submitting or note N/A.

Topic	Item No.	Guide Questions/Description	Reported on Page No.
**Domain 1: Research team and reflexivity**			
Personal characteristics			
Interviewer/facilitator	1	Which author/s conducted the interview or focus group?	
Credentials	2	What were the researcher's credentials? E.g. PhD, MD	
Occupation	3	What was their occupation at the time of the study?	
Gender	4	Was the researcher male or female?	
Experience and training	5	What experience or training did the researcher have?	
Relationship with participants			
Relationship established	6	Was a relationship established prior to study commencement?	
Participant knowledge of the interviewer	7	What did the participants know about the researcher? e.g. personal goals, reasons for doing the research	
Interviewer characteristics	8	What characteristics were reported about the inter viewer/facilitator? e.g. Bias, assumptions, reasons and interests in the research topic	
**Domain 2: Study design**			
Theoretical framework			
Methodological orientation and Theory	9	What methodological orientation was stated to underpin the study? e.g. grounded theory, discourse analysis, ethnography, phenomenology, content analysis	
Participant selection			
Sampling	10	How were participants selected? e.g. purposive, convenience, consecutive, snowball	
Method of approach	11	How were participants approached? e.g. face-to-face, telephone, mail, email	
Sample size	12	How many participants were in the study?	
Non-participation	13	How many people refused to participate or dropped out? Reasons?	
Setting			
Setting of data collection	14	Where was the data collected? e.g. home, clinic, workplace	
Presence of non-participants	15	Was anyone else present besides the participants and researchers?	
Description of sample	16	What are the important characteristics of the sample? e.g. demographic data, date	
Data collection			
Interview guide	17	Were questions, prompts, guides provided by the authors? Was it pilot tested?	
Repeat interviews	18	Were repeat inter views carried out? If yes, how many?	
Audio/visual recording	19	Did the research use audio or visual recording to collect the data?	
Field notes	20	Were field notes made during and/or after the inter view or focus group?	
Duration	21	What was the duration of the inter views or focus group?	
Data saturation	22	Was data saturation discussed?	
Transcripts returned	23	Were transcripts returned to participants for comment and/or	

For peer review only

Topic	Item No.	Guide Questions/Description	Reported on Page No.
		correction?	
**Domain 3: analysis and findings**			
Data analysis			
Number of data coders	24	How many data coders coded the data?	
Description of the coding tree	25	Did authors provide a description of the coding tree?	
Derivation of themes	26	Were themes identified in advance or derived from the data?	
Software	27	What software, if applicable, was used to manage the data?	
Participant checking	28	Did participants provide feedback on the findings?	
Reporting			
Quotations presented	29	Were participant quotations presented to illustrate the themes/findings? Was each quotation identified? e.g. participant number	
Data and findings consistent	30	Was there consistency between the data presented and the findings?	
Clarity of major themes	31	Were major themes clearly presented in the findings?	
Clarity of minor themes	32	Is there a description of diverse cases or discussion of minor themes?	

Developed from: Tong A, Sainsbury P, Craig J. Consolidated criteria for reporting qualitative research (COREQ): a 32-item checklist for interviews and focus groups. *International Journal for Quality in Health Care*. 2007. Volume 19, Number 6: pp. 349 – 357

Once you have completed this checklist, please save a copy and upload it as part of your submission. DO NOT include this checklist as part of the main manuscript document. It must be uploaded as a separate file.

For peer review only
